# Genome-Wide Identification and Abiotic Stress-Responsive Expression Analysis of the *SOS1* Gene Family in *Gossypium hirsutum* L.

**DOI:** 10.3390/life15121843

**Published:** 2025-11-30

**Authors:** Laraib Iqra, Muhammad Naveed Shahid, Gustavo Caetano-Anollés

**Affiliations:** 1 Department of Botany, Division of Science and Technology, University of Education, Lahore 54770, Pakistan; naveed.shahid@ue.edu.pk; 2 Department of Crop Sciences, C.R. Woese Institute for Genomic Biology, University of Illinois at Urbana-Champaign, Urbana, IL 61801, USA

**Keywords:** drought stress, gene expression, phylogeny, promoter, protein structural modeling, salinity tolerance, upland cotton

## Abstract

The *Salt Overly Sensitive 1* (*SOS1*) gene family encodes plasma membrane Na^+^/H^+^ antiporters essential for ionic homeostasis and salt tolerance in plants. Here, we performed a comprehensive genome-wide characterization of *SOS1* genes in allotetraploid cotton (*Gossypium hirsutum* L.). Fifteen *GhSOS1* genes were identified and found unevenly distributed across the A and D subgenomes, indicating that segmental duplication, rather than tandem duplication, was the major driver of family expansion. Phylogenetic analysis resolved four well-supported clades, revealing deep conservation with dicot homologs from *Arabidopsis thaliana*, *Solanum* species, and *Vigna trilobata*, alongside cotton-specific diversification. Ka/Ks ratios indicated strong purifying selection with limited adaptive divergence. Conserved Na^+^/H^+^ exchanger domains and membrane-spanning architectures were maintained, whereas motif and promoter variation suggested functional specialization. Structural modeling confirmed typical multi-helical antiporter topology but revealed the absence of a cytoplasmic regulatory domain, implying alternative modes of regulation, possibly via oxidative stress–response components such as RCD1. Promoter analysis uncovered multiple stress- and hormone-responsive cis-elements, and expression profiling identified *GhSOS1-5* and *GhSOS1-11* as strongly induced by salt and drought stress. Collectively, these findings highlight the evolutionary retention, structural conservation, and regulatory diversification of *GhSOS1* genes, establishing a foundation for improving abiotic stress resilience in cotton.

## 1. Introduction

Cotton, an economically significant crop and the primary natural fiber, forms the backbone of the global textile industry. However, the yield of upland cotton (*Gossypium hirsutum* L.) is greatly compromised by drought and salt stress, both of which cause substantial yield losses. Disruption of ion homeostasis is a key physiological indicator of abiotic stress in *G. hirsutum*.

Drought is one of the major limiting factors in the growth and productivity of upland cotton [1]. Under drought conditions, cotton plants undergo a cascade of physiological changes, including reduced stomatal conductance and a decline in photosynthetic efficiency [2,3]. Spontaneous elevation in reactive oxygen species (ROS) levels is a notable hallmark of drought stress, resulting in cellular damage in plants [4,5]. In addition, drought alters carbohydrate concentrations in leaves and triggers osmotic adjustment mechanisms to maintain cellular homeostasis in *G. hirsutum* [6].

Salt stress is another major challenge to sustainable agriculture, directly reducing the growth and yield of upland cotton [7]. Globally, nearly one-fourth of the agricultural land is affected by salinity, compromising soil integrity and productivity [8]. Upland cotton, along with staple crops such as wheat and rice, is particularly vulnerable to saline irrigation water [9], necessitating the development of tolerance mechanisms and mitigation strategies [10]. Typically, plants exposed to salinity exhibit an increase in root length, while other physiological parameters–including stem diameter, leaf area, and chlorophyll *a* and *b* contents–tend to decline [11].

Traditional practices to mitigate soil salinity, such as soil drainage, soil leaching, and strip cropping, have shown only limited success [12]. Salinity tolerance, on the other hand, is an intricate phenomenon regulated by diverse biochemical, physiological, and molecular responses [13]. Plants employ different mechanisms to tolerate salinity stress, including various tolerance pathways [14]. The most prominent of these is the *salt overly sensitive* (SOS) signaling pathway, which maintains ion equilibrium. This pathway was first characterized in *Arabidopsis thaliana* and comprises three core components: a SOS1 Na^+^/H^+^ transporter, a SOS2 kinase, and a SOS3 Ca^2+^ sensor. SOS1 embodies a central plasma membrane-localized Na^+^/H^+^ exchanger protein responsible for extruding excessive Na^+^ from xylem parenchyma cells [15]. The SOS pathway plays a crucial role in ion homeostasis in cotton under abiotic stress [16]. The *SOS* gene family is evolutionarily conserved and maintains low intracellular Na^+^ levels relative to the extracellular environment, thereby mitigating salt stress [17]. Although *SOS1* homologs have been reported in species such as *Solanum tuberosum*, *Zea mays,* and *Oryza sativa* [18,19], genome-wide investigations in *G. hirsutum* remain necessary.

In recent years, genome-wide studies have become powerful approaches for analyzing gene families and elucidating their expression patterns under abiotic stress [20]. Here, we focus on in silico characterization and expression analysis of the *GhSOS1* gene family through comprehensive genome-wide characterization and functional study. To validate profiling results, quantitative real-time PCR (qRT-PCR) was performed on selected genes to assess expression levels. Considering the economic importance of cotton, these findings contribute to the molecular basis for developing stress-resistant cultivars.

## 2. Materials and Methods

### 2.1. Selection of GhSOS1 Proteins from G. hirsutum

Genomic databases of *G. hirsutum* TM-1 were retrieved from the publicly available Phytozome database [21]. Candidate genes were shortlisted by BLASTP (https://phytozome-next.jgi.doe.gov/blast-search) (all links in the manuscript accessed on 27 November 2025), searched using BLAST against query sequences with a standard parameter e-value of 1 × 10^−5^ and an identity of more than 90%. Query sequences of *SOS1* were retrieved from the model plant *A. thaliana*; protein sequences were obtained from the Arabidopsis Information Resource (TAIR) (https://www.arabidopsis.org/) [22]. The identified protein sequences were subjected to NCBI Batch CD-search to identify the conserved domains present in *GhSOS1* using default parameters (https://www.ncbi.nlm.nih.gov/Structure/bwrpsb/bwrpsb.cgi) [23] and verified using the Pfam (http://pfam.xfam.org/) [24] and SMART (http://smart.embl-heidelberg.de/) [25] databases with default settings. Proteins lacking the Na^+^/H^+^ exchanger domain were discarded.

### 2.2. Phylogenetic Tree Reconstruction and Selection Pressure Analysis

To infer the evolutionary relationships among candidate GhSOS1 proteins and similar proteins from other plant species, a phylogenetic tree was reconstructed with the maximum-likelihood method with 1000 bootstrap replicates in MEGA v11.0.13 (https://www.megasoftware.net/) [26]. The resulting tree was saved in Newick format and exported to the online iTOL tool (https://itol.embl.de/upload.cgi) for graphical visualization. To assess selection pressure among candidate genes’ paralogs, nonsynonymous (Ka) to synonymous (Ks) substitution (Ka/Ks) ratios were calculated using MEGA to evaluate functional divergence and duplication events within gene pairs.

### 2.3. Gene Structure and Motif Analysis

Gene structure was graphically analyzed using TBtools v2.313 with the *G. hirsutum* GFF annotation file method [27]. Conserved domains were identified using Pfam, and motifs in the candidate genes were determined using the MEME Suite (https://meme-suite.org/).

### 2.4. Physicochemical Properties, Subcellular Localization, and Chromosomal Distribution

In silico characterization of the 15 proteins, including molecular weight (KDa), coding sequence (CDS) length (bp), isoelectric point (pI), and grand average of hydropathicity (GAVY), was performed using ProtParam Expasy (https://web.expasy.org/protparam/) [28]. Sub-cellular localization of the candidate proteins was predicted using CELLO v2.5 (http://cello.life.nctu.edu.tw/) and WoLF PSORT (https://wolfpsort.hgc.jp/). The chromosomal distribution of the 15 *GhSOS1* genes was visualized using the online tool Mapgene2Chromosome v2 (http://mg2c.iask.in/mg2c_v2.0/).

### 2.5. AlphaFold2 Structural Predictions and DALI Structural Alignments

Atomic structures were predicted directly from amino acid sequences using the template-based modeling program Phyrev2.2 (https://www.sbg.bio.ic.ac.uk/~phyre2/html/page.cgi?id=index) [29] and the deep learning AlphaFold2 pipeline implemented in ColabFold (https://colab.research.google.com/github/sokrypton/ColabFold/blob/main/AlphaFold2.ipynb). Predicted structures were compared to each other using the DALI server (http://ekhidna2.biocenter.helsinki.fi/dali/) [30], reporting structural similarity dendrograms and hierarchically clustered similarity matrices of Z-scores. Structural alignments and visualizations were carried out using Chimera [31].

### 2.6. Prediction of Phosphorylation Sites and Transmembrane Domains

Phosphorylation sites in GhSOS1 proteins were predicted using the online tool NetPhos 3.1a (https://services.healthtech.dtu.dk/services/NetPhos-3.1/), and transmembrane domains were identified using the bioinformatics tool TMHMM v2.0 (https://services.healthtech.dtu.dk/services/TMHMM-2.0/).

### 2.7. Cis-Regulatory Element Analysis

To identify stress-responsive transcription factors and other cis-regulatory elements in the promoter sequences (2 kb upstream) of the 15 genes, the PlantCARE database was used (https://bioinformatics.psb.ugent.be/webtools/plantcare/html/).

### 2.8. In Silico Expression Profiling and Candidate Gene Selection

Transcript abundance of the SOS1 gene family under abiotic stress was analyzed using publicly available RNA-seq datasets of *G. hirsutum* (Project accession no. PRJNA490626) [32]. The original dataset includes treatments that subjected four-week-old seedlings to salt stress (400 mM NaCl), drought-mimicking stress [20% polyethylene glycol-6000 (PEG)], low temperature (4 °C), and high temperature (37 °C). For the present study, expression values for *GhSOS1* genes were extracted for selected time points (0, 3, 6, and 12 h) under salt and PEG treatments to investigate early stress responses in silico. Gene expression was retrieved and compared across stress treatments and tissues using normalized expression values (FPKM). TBtools was used to compile and visualize these values as a heatmap. Five *GhSOS1* genes that exhibited substantial induction and consistent differential expression patterns under salt and drought conditions were selected for experimental validation using qRT-PCR. The rest of the *GhSOS1* genes, which showed below-average expression and little to no response to treatments, were not explored.

### 2.9. RNA Extraction and cDNA Synthesis

Cotton seedlings of local variety were grown under controlled environmental conditions, including a light intensity of 200 µmol m^−2^ s^−1^, a 16 h light/8 h dark photoperiod, and a day/night temperature regime of 28 ± 2 °C. To impose abiotic stress, one set of 5–7 leafed seedlings was exposed to 400 mM NaCl (heavy salt stress). The NaCl concentration (400 mM) was selected because previous cotton studies commonly use 400 mM NaCl to impose severe salt stress during the seedling stage, reliably activating early salt-responsive pathways such as SOS signaling [33,34]. Another set was treated with 20% (*w*/*v*) PEG-6000 (drought stress). PEG is widely used in laboratory assays because it imposes a stable, non-penetrating osmotic stress that mimics early water-deficit conditions without introducing ionic toxicity. The treatment was applied to induce a well-defined osmotic challenge at the seedling stage for expression profiling. Leaves were harvested at 0 h (control), 3 h, 6 h, and 12 h, with three biological replicates per time point. Total RNA was extracted from 100 mg of fresh leaf tissue using the TRIzol reagent method [35], followed by manual purification [36]. RNA was precipitated with 500 µL isopropanol. The RNA pellet was briefly air-dried and then resuspended in RNase-free water for further use. RNA purity and concentrations were assessed using a Nanodrop spectrophotometer. RNA integrity was first examined on non-denaturing agarose gels and subsequently confirmed under standard analytical conditions using spectrophotometric ratios and electrophoretic assessment. First-strand cDNA was synthesized using the Thermo Scientific RevertAid First Strand cDNA Synthesis Kit, employing a mixture of oligo(dT) primers and random hexamers to ensure complete reverse transcription. Diluted cDNA samples were stored at −80 °C for subsequent qRT-PCR analysis.

### 2.10. Gene Expression Analysis (qRT-PCR) of GhSOS1

Gene expression of selected *GhSOS1* genes was analyzed using the SYBR Green ReadyMix—Fast SYBR Green qRT-PCR system. Primer sequences are listed in Table S1. The amplification protocol was as follows: initial denaturation at 95 °C for 3 min, followed by 40 cycles of 95 °C denaturation, annealing at 55 °C for 30 s, and extension [37]. Gene expression levels were calculated by the 2^−ΔΔCT^ method [38], with the ubiquitin (*GhUBQ*) gene serving as a housekeeping gene. Three biological replicates harvested at different time points were used for each reaction, along with three technical replicates per sample. Statistical significance of expression differences among treatments and time points was assessed with one-way analysis of variance (ANOVA). Tukey’s post hoc test (*p* < 0.05) was performed following ANOVA. Results were presented as mean ± standard error in bar graphs using R software.

## 3. Results

### 3.1. Identification of GhSOS1 Genes and Their Chromosomal Distribution

**Figure 1:**
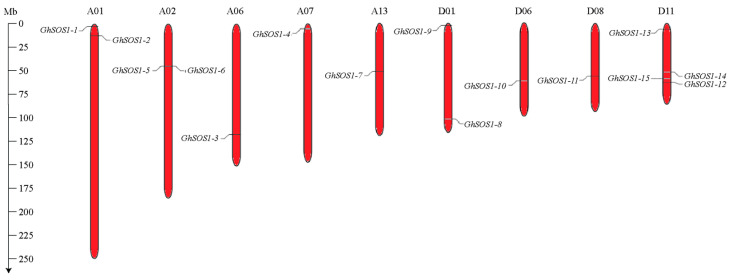
Chromosomal location of *GhSOS1* genes in the *G. hirsutum* genome. The chromosomal distribution of gene locations across nine different chromosomes was determined with the Mg2c tool, with distances measured in megabases (Mb).

Upland cotton is an allotetraploid species (2n = 4x = 52) that originated in Mesoamerica and the Caribbean through hybridization between two ancestral diploid species, an A-genome donor related to the African-Asian species *G. arboreum* (2n = 26), and a D-genome donor related to the American species *G. raimondii* (2n = 26). The chromosomes of the A subgenome are designated A1–A13, and those of the D subgenome are D1–D13. We identified a total of 15 candidate *GhSOS1* gene products in *G. hirsutum* through BLASTP searches, followed by confirmation of their Na^+^/H^+^ exchanger domains using the NCBI Batch CD-Search tool and verification with the Pfam and SMART databases. Chromosomal mapping using the Mg2C tool revealed a non-random distribution of the *GhSOS1* genes across the A and D subgenomes of *G. hirsutum*, suggesting that segmental duplication events contributed to the expansion of this gene family (Figure 1). Specifically, *GhSOS1-1* to *GhSOS1-7* mapped to 5 chromosomes of the A subgenome, while *GhSOS1-8* to *GhSOS1-15* were located on 4 chromosomes of the D subgenome. Most chromosomes contained only one *GhSOS1* gene, except for A01, A02, and D01, which contained two, and D11, which contained four.

### 3.2. Phylogenetic Reconstructions and Selection Pressure Analysis

**Figure 2:**
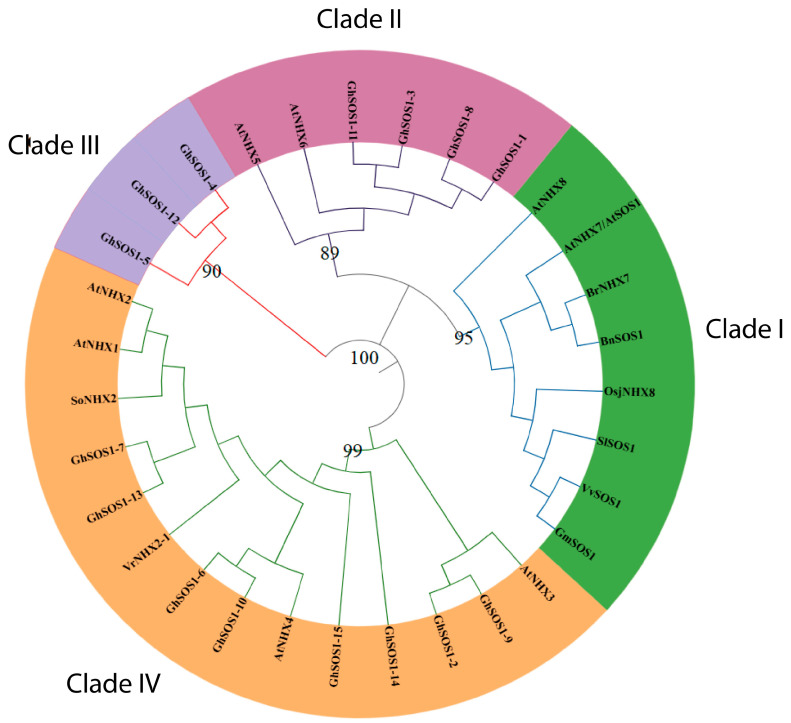
Phylogeny of *GhSOS1* with other monocot and dicot plants. A maximum likelihood (ML) tree was reconstructed with 1000 bootstrap replicates using the JTT model that retained the highest log likelihood (−23,615.62).

To investigate the evolutionary relationships among the 15 GhSOS1 proteins and homologs in reference plant species, a phylogenetic tree was reconstructed using the maximum-likelihood method. Protein sequences in FASTA format are provided in Table S2. The resulting phylogeny resolved four different clades (Figure 2). Clade I contained SOS1 homologs of *O. sativa*, *Vitis vinifera*, *Glycine max,* and *Brassica* species and plasma membrane NHX antiporters from *Arabidopsis thaliana*, but no *G. hirsutum* members, indicating an independent ancestral lineage for these homologs. Clade II, which was sister to Clade I, comprised GhSOS1 members together with *Arabidopsis* endosomal AtNHX5 and AtNHX6 antiporters, reflecting conserved relationships among dicot species. Clade III consisted exclusively of GhSOS1 proteins, representing a lineage-specific group likely derived from recent gene duplication events within the cotton genome. Clade IV included several GhSOS1 proteins clustered with vacuolar antiporters of dicot plants (AtNHX1, AtNHX2, AtNHX3, and AtNHX4, SoNHX2, and VtNHX2-1) that are critical for salt tolerance, suggesting close evolutionary proximity between cotton and *A. thaliana*, *Solanum* species, and *Vigna trilobata* homologs (all dicot plants). Interestingly, the plasma membrane antiporters of *A. thaliana* (AtNHX7/SOS1 and AtNHX8), which play a central role in salt tolerance, were part of Clade I. High bootstrap support across all clades confirmed the robustness of the phylogenetic grouping and underscored both conserved and divergent evolutionary patterns within the *G. hirsutum* SOS1 gene family. Notably, all terminal subclades containing *G. hirsutum* protein pairs included members from both the A and D subgenomes, suggesting that these paralogs originated prior to the ancient hybridization event that gave rise to allotetraploid cotton.

To assess the selective forces acting on the *GhSOS1* gene family, we calculated the Ka/Ks substitution ratios for all identified paralogous gene pairs. This analysis provided insights into the evolutionary constraints influencing these genes and their potential roles in stress responses. Ka/Ks values determined using MEGA showed most of the proteins exhibited ratios less than 1, indicating strong purifying (negative) selection, indicative of functional conservation (Table S3). This suggests that most nonsynonymous mutations are deleterious and are being actively removed by natural selection, preserving the functional integrity of these genes. Only two gene pairs displayed ratios greater than 1, but close to 1, indicating diversifying (positive) selection. This could indicate genes are evolving either neutrally as redundant copies or are the subject of mild adaptive evolution, where advantageous mutations are being favored, potentially enhancing the plant’s ability to cope with abiotic stresses like drought and salinity. Segmental types of duplication events were observed for most *GhSOS1* genes, while four exhibited conserved tandem duplication events.

### 3.3. Analysis of Conserved Domains, Motifs, and Gene Structures

**Figure 3:**
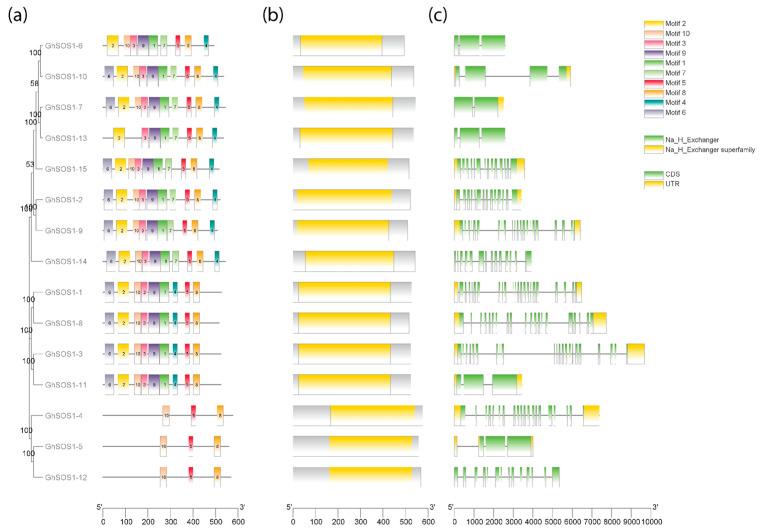
Conserved motif (**a**) and domain (**b**) analysis of the GhSOS1 proteins and corresponding gene structures detected across protein sequences (**c**). Conserved motifs are represented by distinct colored boxes, and the Pfam Na^+^/H^+^ exchanger (PF00999) domains are identified as yellow boxes. *GhSOS1* gene structures show coding sequences (CDSs) and untranslated regions (UTRs) as green and yellow boxes, respectively, and introns as lines. The maximum likelihood tree on the left was reconstructed with 1000 bootstrap replicates using the JTT model (log likelihood = −16,861.58).

All 15 GhSOS1 proteins contained Na^+^/H^+^ exchanger domains, although the length and arrangement of Pfam domain structures varied across protein sequences (Figure 3). Motif analysis using the MEME statistical model tool identified 10 conserved motifs represented as ungapped, position-dependent letter-probability matrices among the unaligned sequences. Of these, Motifs 5, 8, and 10 were consistently present in all GhSOS1 proteins. Their universal occurrence and conserved alignment indicate strong functional conservation and suggest that these motifs constitute a core structural module, likely associated with the essential Na^+^/H^+^ exchanger activity.

A clear association was observed between motif composition and the phylogenetic clades in the reconstructed phylogeny of Figure 2. Most clade members displayed a rich repertoire of motifs. However, the GhsOS1-4, GhSOS1-5, and GhSOS1-12 proteins of Clade III contained only Motifs 5, 8, and 10, implying substantial sequence divergence from other paralogs. Similarly, GhSOS1-1, GhSOS1-3, GhSOS1-8, and GhSOS1-11 of Clade II were uniquely defined by the absence of Motif 4, which represented a structural signature specific to this group. In contrast, the eight members of Clade IV shared a unique signature motif, Motif 4, although some variation was observed: GhSOS1-13 lacked Motifs 6 and 10, while GhSOS1-6 was distinguished from its clade members by the absence of Motif 6.

The clade-specific distribution and loss of motifs suggest potential functional diversification among *GhSOS1* paralogs, possibly reflecting gene-specific regulatory adaptations or structural modifications underlying differential responses to abiotic stress.

In contrast with their encoded proteins, *GhSOS1* genes displayed substantial variation in length and in the number of exons and introns present along their coding sequences (CDSs) (Figure 3). Several genes possessed relatively large untranslated regions (UTRs), particularly at the 3′ end. These UTRs are known to regulate translation initiation and termination, influence mRNA stability and localization, and contain binding sites for microRNAs or RNA-binding proteins. The observed diversity in UTR size and structure suggests that post-transcriptional regulation may play an important role in modulating *GhSOS1* gene expression.

### 3.4. Physicochemical Properties and Protein Sub-Cellular Localization

**Figure 4:**
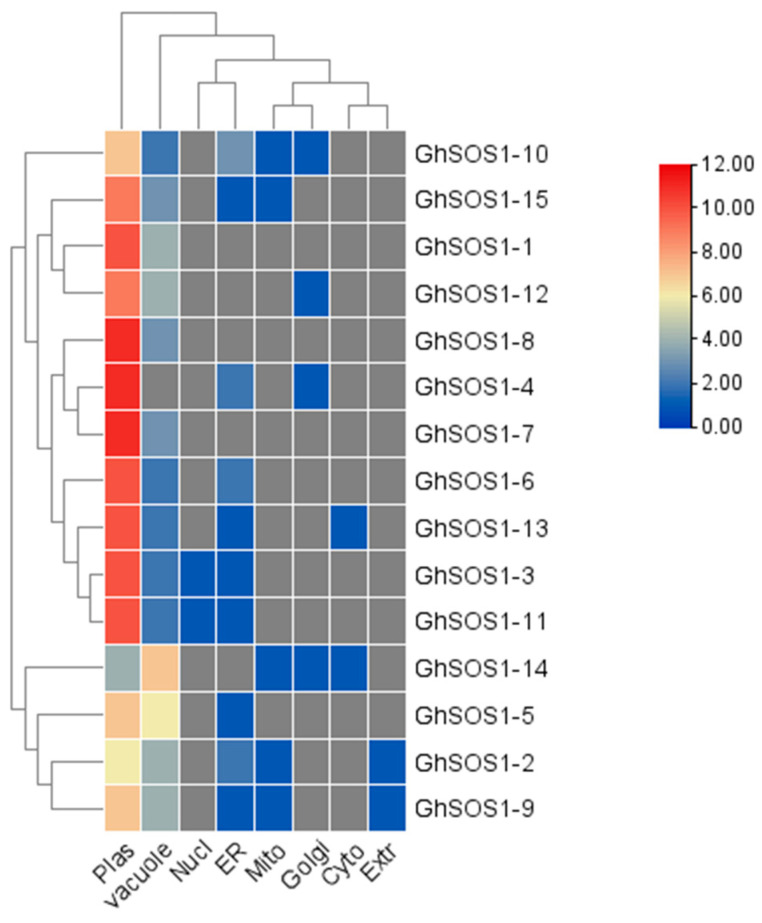
Predicted subcellular localization of GhSOS1 proteins. Heatmap showing predicted subcellular localization across different cellular compartments. Color intensity represents prediction confidence.

Molecular weights and isoelectric points of GhSOS1 proteins ranged from 56.8–90.5 kDa and 5.35–8.99, respectively, inducing significant changes in protein solubility. Amino acid counts ranged from 515–575 residues. The CDS length of their genes ranged from 774–1800 bps (Table 1). Most of the GhSOS1 proteins were localized in the plasma membrane (Figure 4).

### 3.5. Structural Predictions, Transmembrane Topologies, and DALI Structural Alignments

**Figure 5:**
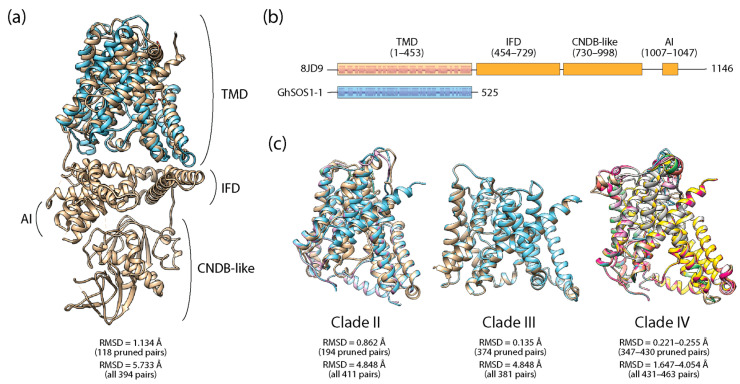
Structural alignment of a Phyre 2.2-predicted *GhSOS1-1* structure (blue backbone) against the published structure of a SOS1 Na^+^/H^+^ antiporter of *A. thaliana* (PDB entry 8JD9 in tan) (**a**), schematic plot of sequence and structure alignments (**b**), and alignments of Phyre 2.2-predicted *GhSOS1* structures of individual clades (**c**). TMD, transmembrane domain; IFD, interfacial domain; CNDB-like, cyclic-nucleotide-binding domain; AI, autoinhibition domain.

We predicted the atomic structure of GhSOS1 proteins directly from amino acid sequences using both the template-based modeling program Phyre 2.2, which yields reliable results when homologous templates are available (including those from the AlphaFold database), and the State-of-the-Art deep learning-based AlphaFold2 ab initio pipeline, which achieves near-experimental accuracy in structural prediction. Both approaches produced highly consistent models (TM-scores = 0.563–0.806) of high confidence (Table S4), indicating that remote homology detection can achieve accuracy comparable to that of deep learning methods when suitable antiporter templates of known structure are available. Structural alignments between the predicted models and experimentally determined SOS1 proteins from other plant species further supported the validity of predictions and confirmed the conserved role of GhSOS1 proteins in ion transport. As shown in Figure 5a, the Phyre 2.2-predicted GhSOS1-1 protein structure aligned closely with the SOS1 Na^+^/H^+^ antiporter of *A. thaliana* (PDB entry 8JD9), exhibiting acceptable RMSD values across the transmembrane domain (TMD). However, the *G. hirsutum* protein lacked the large cytoplasmic domain (CPD) that modulates Na^+^/H^+^ exchange activity in *Arabidopsis* (Figure 5b), which encompasses a regulatory interfacial domain, an autoinhibition domain, and an activating CNDB-like basal domain [39]. Similar alignment results were obtained for the rest of the 15 GhSOS1 proteins. Table S5 shows a sequence and structural alignment (TM-score = 0.825), showing the absence of CPD in GhSOS1-1.

Alignment between predicted structures of members of each phylogenetic clade revealed both remarkable structural conservation and notable structural diversity (Figure 5c). Clade III proteins exhibited simpler architectures and aligned almost perfectly with one another, reflecting high structural conservation. In contrast, members of Clade II and IV displayed progressively greater structural variability, consistent with the observed trends and motif variability within their corresponding protein sequences.

**Figure 6:**
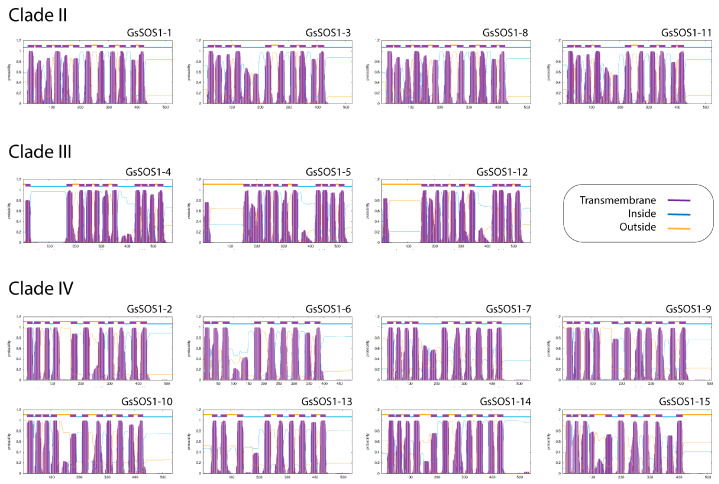
TMHMM posterior probabilities along the sequences of GhSOS1 protein members of phylogenetic clades, indicating interactions of α-helices with membrane (transmembrane), cytosolic (inside), and external (outside) environments.

GhSOS1 proteins exhibited the typical multi-helical structure of Na^+^/H^+^ antiporters that is critical for cation exchange, with an overall design that was relatively conserved. Prediction of the transmembrane topology of proteins using the TMHMM tool confirmed they function as integral membrane proteins (Figure 6). The analysis predicted multiple alternating regions embedded within the membrane (transmembrane α-helical domains) flanked by regions exposed to the cytoplasmic (inside) or external (outside) environments across the entire protein sequence. Remarkably, alternating regions followed patterns that again placed proteins into three distinct groups, matching their membership in Clades II, III, and IV identified in maximum likelihood phylogenetic reconstructions.

**Figure 7:**
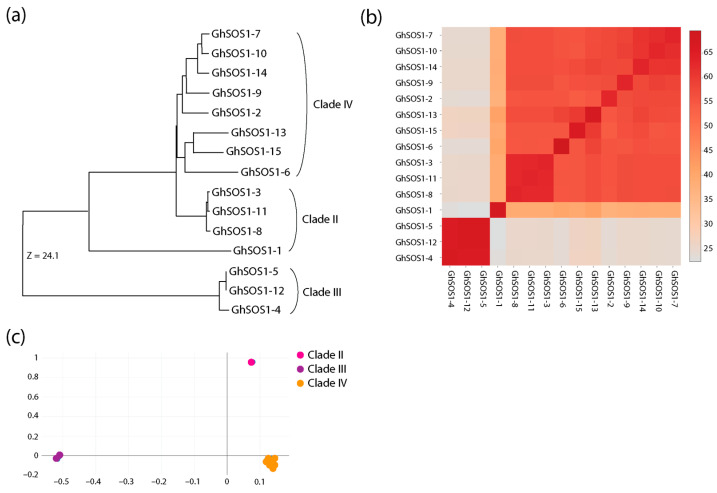
Structural similarity dendrogram (**a**), hierarchically clustered similarity matrix of Z-scores (**b**), and projections of the multidimensional scaling method of correspondence analysis (**c**) of all-against-all pairwise DALI structural alignments of Phyre2.2-predicted GhSOS1 proteins.

To compare the predicted structures to each other, we used the distance-matrix alignment method for all-against-all pairwise comparisons of the DALI server. Structural similarity dendrograms, similarity matrices of Z-scores, and correspondence analyses pooled structures into groups that matched clades previously visualized in maximum likelihood reconstructions. Members of Clade III (GhSOS1-4, GhSOS1-5, and GhSOS1-12) formed a group at the base of the dendrogram, followed by members of Clade II (GhSOS1-1, GhSOS1-3, GhSOS1-8, and GhSOS1-11), and members of Clade IV (the rest of the GhSOS1 proteins), in that order (Figure 7). AlphaFold2-predicted structures were similarly grouped to those predicted by Phyre 2.2 (Figure S1).

### 3.6. Prediction of Phosphorylation Sites

Phosphorylation of specific amino acid residues can activate or inactivate proteins by inducing conformational changes, thereby modulating their function. These modifications can also promote or disrupt binding to other proteins, mark proteins for ubiquitination and proteosomal degradation, enable signal transduction cascades, or determine subcellular localization. Putative phosphorylation sites of GhSOS1 proteins were identified with Net Phos 3.1a, a neural network-based predictor trained to recognize sequence motifs and local amino acid environments characteristic of phosphorylation. The analysis revealed multiple potential phosphorylation sites, suggesting that post-translational modification may provide a versatile regulatory mechanism modulating GhSOS1 antiporter activity in response to environmental cues such as drought and salinity stress. Phosphorylation sites were unevenly distributed across the protein sequences, with serine residues being modified more frequently than threonine or tyrosine residues (Figure S2).

### 3.7. Promoter Region Analysis and Cis-Regulatory Elements

**Figure 8:**
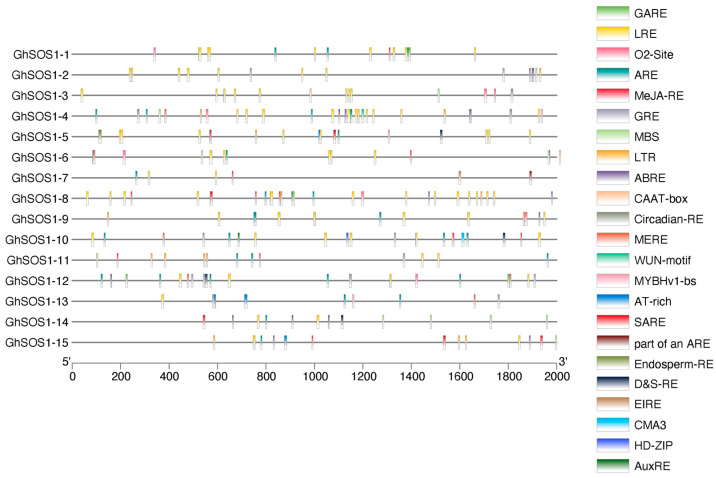
Promoter regions of *GhSOS1* genes show multiple stress- and hormone-responsive motifs.

Promoter analysis identifies conserved cis-regulatory motifs located upstream of genes that serve as binding sites for transcription factors. These elements are critical for predicting transcriptional regulation, comparing promoter architectures across gene families, and guiding functional validation of gene expression patterns. Multiple cis-regulatory elements were detected in promoter regions of *GhSOS1* genes, indicating their potential involvement in drought and salinity stress tolerance in cotton (Figure 8).

The *GhSOS1* promoters contained a diverse array of stress- and hormone-responsive cis-elements, forming a complex regulatory network that modulates gene expression in response to environmental cues such as drought, salinity, temperature stress, and phytohormone signaling. Abiotic stress-responsive elements included the anaerobic response element (ARE), which mediates transcription under low oxygen conditions and responds to ROS accumulation during hypoxia-reoxygenation cycles; the low temperature-responsive element (LTR) which often integrates ROS-dependent transcriptional activation and is associated with cold-inducible expression; the MYB-binding site (MBS), involved in drought and dehydration responses as well as ROS homeostasis; the MYBHv1-binding site, which mediates responses to drought, salinity, cold stress and ROS detoxification; the WUN-motif, which regulates wound-responsive genes that often elicit ROS-mediated signaling cascades; and the light-responsive element (LRE), which participates in general stress and light-regulated expression. LTR and LRE were particularly abundant in all promoter regions, while others were present in promoter subsets. For example, MBSs were present in *GhSOS-1*, *GhSOS-3*, *GhSOS-4*, *GhSOS-6*, *GhSOS-11*, *GhSOS-12*, *GhSOS-14* (3 copies), and *GhSOS-15*.

Hormone-responsive elements included the ABA-responsive element (ABRE), involved in abscisic acid-mediated responses to drought, salinity, oxidative stress, and seed development; the auxin-responsive element (AuxRE), regulating auxin-dependent growth and stress signaling; and the GA-responsive element (GARE), implicated in gibberellin-dependent growth and development regulation. The abundance of these elements was low and restricted to some promoters.

### 3.8. In Silico Expression Profiling and Candidate Gene Selection

**Figure 9:**
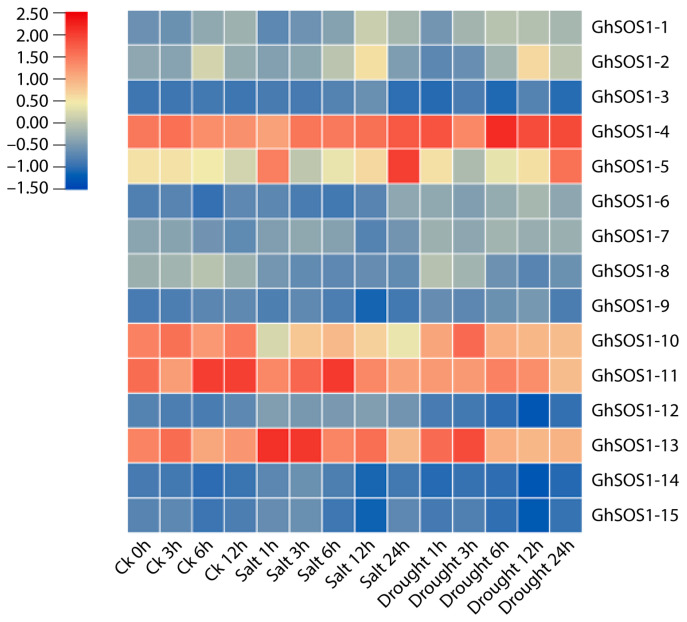
Heatmap of *GhSOS1* genes showing log_2_-normalized RNA-seq expression under salt and drought stress (0–24 h). Genes *GhSOS1*-4, *GhSOS1*-5, *GhSOS1*-10, *GhSOS1*-11, and *GhSOS1*-13 displayed consistent stress-responsive patterns and were therefore selected for qRT-PCR validation. The colors scale describes row-wise Z-score–standardized log_2_(FPKM) expression values.

To investigate the transcriptional responses of *GhSOS1* genes to abiotic stress, we analyzed their expression profiles under salt and drought conditions using RNA-seq data retrieved from publicly available cotton transcriptome datasets (Project: PRJNA490626). Expression levels of all *GhSOS1* genes were quantified at 0, 3, 6, 12, and 24 h post-treatment, normalized, and visualized as a heatmap to reveal temporal expression dynamics under stress conditions. Members of the *GhSOS1* gene family displayed distinct expression patterns throughout treatments or relative to the control (Figure 9). Most members showed below-average expression (blue hues in the heatmap) and little to no response to treatments, suggestive of low responsiveness or possible tissue-specific roles not captured in the sampled tissue. In contrast, genes *GhSOS1-4*, *GhSOS1-5*, *GhSOS1-10*, *GhSOS1-11*, and *GhSOS1-13* exhibited clear upregulation (red hues in the heat map), and with the exception of *GhSOS1-11*, control treatments remained mostly neutral, showing stable expression. However, responses varied under salt or drought stress. *GhSOS1-4* responded weakly to salt at 24 h and strongly to drought, especially at 6 h. *GhSOS1-5* responded strongly to both salt and drought stress at 24 h but remained mostly neutral under other treatments. *GhSOS1-10* showed weak downregulation to salt at all time intervals, while *GhSOS1-11* showed upregulation to salt at 6 h. Finally, *GhSOS1-13* showed upregulation to salt at 1 h and 2 h and to drought at 3 h. The expression of this set of genes is consistent with induction under stress, typical of genes involved in stress tolerance. Owing to their stress-responsive expression profiles, these genes were selected for experimental validation.

### 3.9. Gene Expression Analysis (qRT-PCR) of GhSOS1 Genes

**Figure 10:**
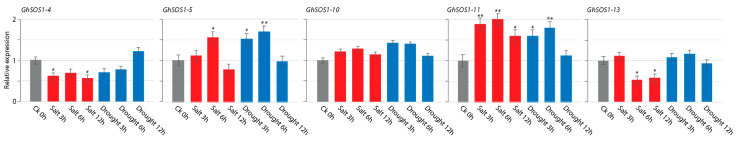
Gene expression analysis. Reactions were performed in triplicate biological replicates, and mean expression values were statistically analyzed using ANOVA followed by Tukey’s post hoc test (*p* < 0.05). Bar graphs represent mean ± standard error, with asterisks (* *p* < 0.05; ** *p* < 0.01) above each bar indicating statistically significant treatment- and time-specific differences.

To validate the RNA-seq results and further examine the transcriptional regulation of *GhSOS1* genes under abiotic stress, qRT-PCR was performed for selected members showing consistent upregulation in in silico expression analyses. Cotton seedlings were subjected to 400 mM NaCl (salt stress) and 20% PEG (drought stress) treatments, and gene expression levels were analyzed at multiple time points (Figure 10).

Distinct temporal responses were observed across treatments, some of which were consistent with in silico RNA-seq predictions. Under salt stress, *GhSOS1-5* and *GhSOS1-11* exhibited marked up-regulation at early (3 h) and mid (6 h) stages, indicating rapid and sustained stress responsiveness. Likewise, *GhSOS1-11* maintained elevated transcript levels under drought conditions, suggesting a broader role in osmotic stress tolerance. These patterns were generally consistent with RNA-seq expression profiling. In contrast, *GhSOS1-4* and *GhSOS1-13* displayed down-regulation with significantly reduced transcript abundance under salinity, which contradicts RNA-seq predictions. Meanwhile, *GhSOS1-10* remained largely unchanged, displaying stable expression throughout all treatments and time intervals, again differing from RNA-seq data. We attribute the discrepancies between qRT-PCR and RNA-seq results to unaccounted variation in plant growth or stress-induction conditions between experiments.

## 4. Discussion

The identification and chromosomal localization of *GhSOS1* genes in *G. hirsutum* highlight the evolutionary complexity and genomic organization of this gene family in polyploid cotton. The uneven distribution of *GhSOS1* members across the A and D subgenomes suggests that segmental duplication rather than tandem duplication played a predominant role in their expansion. This pattern is consistent with previous observations in other polyploid plant species, where gene retention following whole-genome duplication contributes to functional diversification and sub-functionalization of stress-related genes [40]. The presence of multiple *GhSOS1* copies on certain chromosomes (e.g., D11) may further indicate localized gene amplification or selection pressure favoring redundancy and neofunctionalization in ionic homeostasis and salt tolerance mechanisms.

To unfold an evolutionary framework for understanding how SOS1-mediated Na^+^/H^+^ exchange systems diversified within the allopolyploid genome of *G. hirsutum* to enhance its adaptability to salinity and other abiotic stresses, phylogenetic and selection pressure analyses were conducted. Maximum-likelihood phylogeny resolved four well-supported clades, revealing both deep evolutionary conservation and lineage-specific expansion. The clustering of several GhSOS1 proteins with *A. thaliana*, *S. lycopersicum,* and *V. trilobata* protein homologs indicates that these Na^+^/H^+^ antiporters share a conserved evolutionary lineage among dicot species, reflecting retention of the core Na^+^/H^+^ exchanger domains across eudicots. In contrast, the cotton-specific Clade III, composed exclusively of GhSOS1 members, implies that post-polyploidization duplication—likely segmental—enabled sub-functionalization or neofunctionalization within *G. hirsutum*. The inclusion of both A- and D-subgenome members in terminal subclades further supports the hypothesis that these duplications predated the allotetraploidization event.

This pattern mirrors that observed in other polyploid plant species, where gene retention following whole-genome duplication contributes to functional diversification and sub-functionalization of stress-related genes [40,41]. Such retention of duplicated *SOS1* paralogs in both subgenomes likely provides an evolutionary advantage by maintaining dosage balance and enabling differential regulation under environmental stress.

Ka/Ks ratios further clarified the selective forces shaping GhSOS1 protein evolution. Most paralogous *GhSOS1* gene pairs are under strong purifying selection (Ka/Ks < 1), reflecting evolutionary constraints to preserve essential Na^+^/H^+^ antiporter functions that maintain cellular ion balance and salt tolerance. A few gene pairs showed Ka/Ks ratios slightly above 1, indicating mild positive selection, which may drive functional specialization and adaptive divergence. These mildly diversifying lineages could represent functionally specialized variants acquiring novel or fine-tuned regulatory roles in abiotic stress responses. The observation that most *GhSOS1* duplications were segmental rather than tandem agrees with typical patterns of gene family evolution in polyploid cotton, where segmental duplication following genome merger contributes to long-term retention of stress-related loci. Collectively, the phylogenetic topology, duplication patterns, and selective constraints reveal a dynamic yet functionally constrained evolutionary trajectory of the *GhSOS1* gene family, balancing conservation of core Na^+^/H^+^ exchanger functions with limited stress-responsive divergence. These evolutionary insights can now be translated into crop improvement for fiber quality and stress resilience [42,43].

Motif and domain analyses further support the functional importance of GhSOS1 proteins. All GhSOS1 members contain Na^+^/H^+^ exchanger domains and highly conserved Motifs 5, 8, and 10. Domains and motifs form a core structural module essential for ion transport [44]. Such conserved motifs have been reported in other plant species, and subtle motif variations among paralogs may indicate gene-specific roles in response to abiotic stresses [45,46,47]. These structural features, combined with the physiochemical properties of the proteins, such as hydrophilicity and intracellular stability, confirm the involvement of GhSOS1 proteins in *G. hirsutum* under drought and salinity stress [48], suggesting they are well-equipped to function as integral membrane antiporters involved in xylem sodium unloading and maintenance of cellular ion balance [49]. Moreover, the implication of GhSOS1 proteins in stress-receptor signaling reinforces their functional role in stress adaptation [50]. Segmental duplication has likely contributed to the diversification of *GhSOS1* functions across chromosomes [51], while the presence of cis-regulatory elements in their promoter regions—including auxin-, drought-, and salt-responsive motifs—supports their potential involvement in stress-responsive transcriptional regulation, consistent with observations in other plant species [52].

Predicted three-dimensional atomic structures from Phyre2.2 and AlphaFold2 confirm that GhSOS1 proteins maintain the multi-helical architecture typical of Na^+^/H^+^ antiporters and function as integral membrane proteins. Structural alignment with experimentally determined *A. thaliana* SOS1 proteins validated the models, though *G. hirsutum* proteins lacked the large cytoplasmic regulatory domain (CPD) present in *Arabidopsis* [53]. The absence of a CPD is significant because it suggests these proteins lack a cytoplasmic regulatory control system that senses cytosolic Na^+^ concentrations. Instead, GhSOS1 activities may be regulated by interactions with other proteins outside the canonical SOS1 pathway, such as the oxidative stress-response regulator RCD1 [54], highlighting a potential species-specific mechanism for modulating ion transport under stress.

Clade-level structural comparisons reinforce sequence-based findings: Clade III members exhibit simpler, highly conserved architectures, consistent with their limited motif repertoire and suggesting functional conservation within this clade. In contrast, Clades II and IV show greater structural complexity and variability, paralleling motif and phylogenetic divergence, and suggesting clade-specific functional adaptation. DALI-based structural similarity analyses recapitulate phylogenetic relationships, further emphasizing the co-evolution of sequence, structure, and function.

Post-translational modification and transcriptional regulation appear to act in concert to fine-tune GhSOS1 activity under abiotic stress. Phosphorylation is a central mechanism in stress-responsive signaling, enabling rapid modulation of transporter function in response to ionic and osmotic perturbations. Our identification of multiple serine-rich phosphorylation sites across GhSOS1 proteins suggests that their activity may be dynamically regulated by protein kinases in response to salt and drought stress. Such modifications could influence subcellular localization, interaction with signaling partners, or turnover rates, thereby modulating Na^+^/H^+^ exchange efficiency. This observation is consistent with prior reports highlighting phosphorylation-dependent activation of SOS1 antiporters under salinity stress in other plants [55].

The structural organization of GhSOS1 further reinforces its presumed role in ionic homeostasis and stress tolerance. The predicted presence of multiple transmembrane α-helices, interconnected by intra- and extracellular loops, supports its function as an integral membrane transporter mediating Na^+^ efflux and K^+^/Na^+^ balance. These features, along with conserved Na^+^/H^+^ exchanger domains, mirror those reported in other plant *SOS1* homologs and align with the canonical inhibition–release mechanism that governs *SOS1* activation under salt stress [56,57]. Together, these attributes substantiate the evolutionary conservation of SOS1 function in maintaining cytosolic ion equilibrium and preventing sodium toxicity during osmotic challenge.

Regulation of *GhSOS1* genes at the transcriptional level further underscores their functional integration in stress adaptation. Promoter analysis revealed a diverse suite of cis-regulatory elements responsive to abiotic stresses and phytohormones, including ABRE, ARE, LTR, MBS, AuxRE, and GARE motifs, which mediate transcriptional responses to abscisic acid, low temperature, drought, salinity, oxidative stress, and developmental cues. The coexistence of these motifs suggests that *GhSOS1* transcription is governed by a complex regulatory network that integrates environmental and hormonal signals, paralleling patterns observed in other stress-associated genes [52]. Such promoter diversity likely allows differential spatiotemporal regulation of individual *GhSOS1* paralogs, reflecting sub-functionalization of expression following gene duplication.

Experimental expression profiling provided further evidence of differential regulatory specialization among *GhSOS1* members. Both RNA-seq and qRT-PCR analyses demonstrated distinct temporal and treatment-specific expression patterns under salt and drought stress. Notably, *GhSOS1-5* and *GhSOS1-11* exhibited strong up-regulation at early (3 h) and mid (6 h) time points, suggesting a role in the rapid activation of Na^+^ extrusion and osmotic adjustment pathways. This early transcriptional induction parallels the behavior of *SOS1* orthologs in *A. thaliana* and *O. sativa*, where enhanced expression contributes to maintaining K^+^/Na^+^ ratios and conferring salt tolerance [58,59]. In contrast, *GhSOS1-10* showed stable expression across treatments, indicative of a possible housekeeping or tissue-specific function unrelated to acute stress responses. Meanwhile, the down-regulation of *GhSOS1-4* under salinity mirrors the behavior of certain *SOS1* orthologs in potato and barley, where feedback inhibition may operate to optimize energy expenditure and prevent excessive ion flux [49,60].

Taken together, our genome-wide study not only characterizes the *GhSOS1* repertoire but also reveals a regulatory architecture that is more complex than the canonical SOS pathway described in *Arabidopsis*. In the classical model, SOS1 activity is primarily governed by the SOS2–SOS3 kinase complex, which phosphorylates the regulatory CPD of SOS1 to activate Na^+^ efflux [61]. However, the apparent absence or reduction in a comparable CPD in *G. hirsutum* suggests that cotton may rely on alternative regulatory mechanisms to modulate SOS1 activity. This raises a key biological question: how is SOS1 activation achieved in the absence of a robust CPD-based sensory module?

Our structural predictions and promoter analyses, together with the distribution of phosphorylation sites, point toward a more distributed and protein-interaction–dependent regulatory logic. One particularly compelling candidate is the oxidative stress-response regulator RCD1, which has been shown in other systems to modulate membrane protein activity under ROS-rich stress environments [54]. Although our study did not identify discrete RCD1-interaction motifs within GhSOS1 proteins, the enrichment of ROS/oxidative stress–responsive cis-elements in several GhSOS1 promoters (including ARE, O2-Site, WUN-motif, MBS, ABRE, LTR, SARE, AT-rich, MYBHv1-bs, D&S-RE, and EIRE), the stress-induced transcriptional signatures we observed, and prior evidence that RCD1 modulates the activity of diverse membrane-associated stress proteins collectively support the hypothesis that cotton may have rewired portions of the SOS network to integrate redox cues into ionic homeostasis [62].

Based on these findings, we propose a testable model in which RCD1 functions as a conditional co-regulator of SOS1 activity in *G. hirsutum*. Under salt-induced oxidative stress, RCD1 may influence the activation state, stability, or membrane localization of specific GhSOS1 paralogs, either indirectly through modulation of upstream kinases/phosphatases or directly through protein–protein interactions yet to be demonstrated. This model yields several experimentally tractable predictions: (i) silencing or CRISPR-editing of *GhRCD1* should disproportionately affect the salt-induced up-regulation and function of early-responsive gene paralogs such as *GhSOS1-5* and *GhSOS1-11*; (ii) co-expression correlations between *GhRCD1* and *GhSOS1* paralogs should strengthen under combined ionic and oxidative stress; and (iii) *in vitro* or *in planta* interaction assays may reveal physical associations between RCD1 and selected GhSOS1 proteins, particularly those showing strong oxidative-stress transcriptional responses.

Developing this model places the *GhSOS1* family within a broader integrative signaling context, aligning with expanding knowledge that SOS-mediated ion homeostasis is not solely ion-driven but is cross-regulated by ROS, hormonal signals, and metabolic status. It also provides a mechanistic framework for understanding why cotton retains multiple segmentally duplicated *GhSOS1* copies: differential regulatory wiring—rather than differences in transporter architecture—may underlie sub-functionalization. Future functional validation of this RCD1-centered regulatory hypothesis will be critical for clarifying how cotton coordinates ionic and oxidative stress responses and may offer new leverage points for engineering salt-resilient cultivars.

## 5. Conclusions

This study provides the first comprehensive, genome-wide characterization of the *SOS1* gene family in *G. hirsutum*, revealing how evolutionary history, structural features, and regulatory complexity converge to contribute to salt and drought tolerance. The combined evolutionary, structural, genetic, and functional investigations depict the *GhSOS1* gene family as a finely tuned system that integrates structural conservation, evolutionary retention, and regulatory plasticity to maintain ionic homeostasis under adverse conditions. The preservation of core Na^+^/H^+^ exchanger domains ensures fundamental antiporter functionality, while diversification in motifs, phosphorylation sites, and promoter architectures introduces layers of post-translational and transcriptional control that enable gene- and clade-specific regulation. These structural and regulatory adaptations, together with motif loss, domain variation, and alternative protein interactions, likely allow *G. hirsutum* to fine-tune ionic homeostasis and optimize stress responses under fluctuating salt and drought conditions. Such modular flexibility reflects the dynamic interplay between genome duplication, structural evolution, and functional diversification in polyploid plants [42], harnessing genomic redundancy for environmental resilience.

Expression profiling under salt and drought stress identified significantly upregulated and downregulated *GhSOS1* paralogs, implying differential regulatory roles among family members. These patterns were often consistent with *in silico* predictions and underscore the division of labor among duplicated genes in maintaining ionic equilibrium. However, we note that our study identifies associations rather than demonstrating causality, as we did not perform functional genomic or physiological experiments (e.g., overexpression, knockout/knockdown analyses, ion-flux measurements) that would provide direct evidence for gene function.

From an applied perspective, our findings establish a robust evolutionary and functional framework for understanding Na^+^/H^+^ antiporter regulation in cotton. The differentially expressed *GhSOS1-5* and *GhSOS1-11* genes could serve as key candidates for enhancing abiotic stress resilience through marker-assisted selection or transgenic approaches. Future functional characterization, including protein–protein interaction assays, promoter activity analyses, and identification of upstream kinases, will be key to dissecting the broader SOS regulatory network in polyploid cotton and translating these insights into crop improvement strategies.

## Figures and Tables

**Table 1:** Physiochemical properties of GhSOS1 proteins. Chr, chromosome; +ve, forward strand; −ve, reverse strand; CDS, coding sequence; MW, molecular weight; pI, isoelectric point.

Transcript ID	Gene Name	Start	End	Chr	Strand	CDS (bp)	Protein Length (Residues)	MW (kDa)	pI
Gohir.A01G036500.1.p	*GhSOS1-1*	2,907,644	2,914,117	A01	+ve	1455	525	57,682.83	5.83
Gohir.A01G085600.1.p	*GhSOS1-2*	12,718,712	12,722,123	A01	−ve	1563	520	58,385.64	7.65
Gohir.A06G181100.1.p	*GhSOS1-3*	123,551,254	123,560,921	A06	+ve	1572	523	57,725.56	5.35
Gohir.A07G039800.1.p	*GhSOS1-4*	5,201,392	5,208,768	A07	−ve	1728	575	62,278.06	5.91
Gohir.A02G103100.1.p	*GhSOS1-5*	47,530,475	47,534,471	A02	+ve	2403	557	86,844.88	8.63
Gohir.A02G103200.1.p	*GhSOS1-6*	47,628,984	47,631,749	A02	+ve	774	493	86,783.59	8.70
Gohir.A02G107001.1.p	*GhSOS1-7*	53,796,550	53,799,059	A02	−ve	2115	543	76,359.76	8.99
Gohir.A13G193600.1.p	*GhSOS1-8*	106,866,865	106,874,598	A13	+ve	1800	515	64,659.58	6.03
Gohir.D01G023800.1.p	*GhSOS1-9*	2,596,207	2,602,613	D01	+ve	1344	508	56,877.92	5.51
Gohir.D01G209600.1.p	*GhSOS1-10*	64,539,990	64,545,894	D01	−ve	2484	535	90,258.69	8.17
Gohir.D06G166000.1.p	*GhSOS1-11*	58,829,180	58,832,616	D06	+ve	2472	523	90,511.16	8.49
Gohir.D08G235650.1.p	*GhSOS1-12*	66,082,200	66,087,538	D08	+ve	1722	567	63,860.61	8.09
Gohir.D11G077600.1.p	*GhSOS1-13*	6,530,454	6,533,042	D11	+ve	2361	534	87,643.38	6.43
Gohir.D11G248200.2.p	*GhSOS1-14*	54,223,810	54,227,732	D11	−ve	1605	542	59,734.17	8.64
Gohir.D11G272700.1.p	*GhSOS1-15*	61,187,575	61,191,154	D11	−ve	1629	515	59,787.77	6.00

## Data Availability

RNA-seq data for *GhSOS1* FPKM values can be retrieved from publicly available cotton transcriptome datasets (NCBI Project: PRJNA490626). Predicted structures are deposited in ModelArchive.

## Supplementary Materials

The following supporting information can be downloaded at: https://www.mdpi.com/article/10.3390/life15121843/s1, Table S1: Primer sequences, Table S2: GhSOS1 protein sequences, Table S3: Selection pressure analysis, Table S4: Confidence of Phyre 2.2 and AlphaFold2 predictions and TM-scores of structural alignments, Table S5: Structural alignment of the SOS1 Na^+^/H^+^ antiporter of *A. thaliana* (PDB entry 8JD9) and the GhSOS1-1 protein, Figure S1: Structural similarity dendrogram (a), hierarchically clustered similarity matrix of Z-scores (b), and projections of the multidimensional scaling method of correspondence analysis (c) of all-against-all pairwise DALI structural alignments of AlphaFold2-predicted GhSOS1 proteins, Figure S2: Phosphorylation sites in GhSOS1 proteins predicted using Net Phos 3.1.
