# Supplementary material for: Genome-Wide Identification and Abiotic Stress-Responsive Expression Analysis of the SOS1 Gene Family in Gossypium hirsutum L
Source: Life (Basel). 2025 Nov 30;15(12):1843. doi: 10.3390/life15121843 (PMC12735070; doi:10.3390/life15121843)
Supplement: Supplementary file 1 [file life-15-01843-s001.zip › Figure S1.pdf]

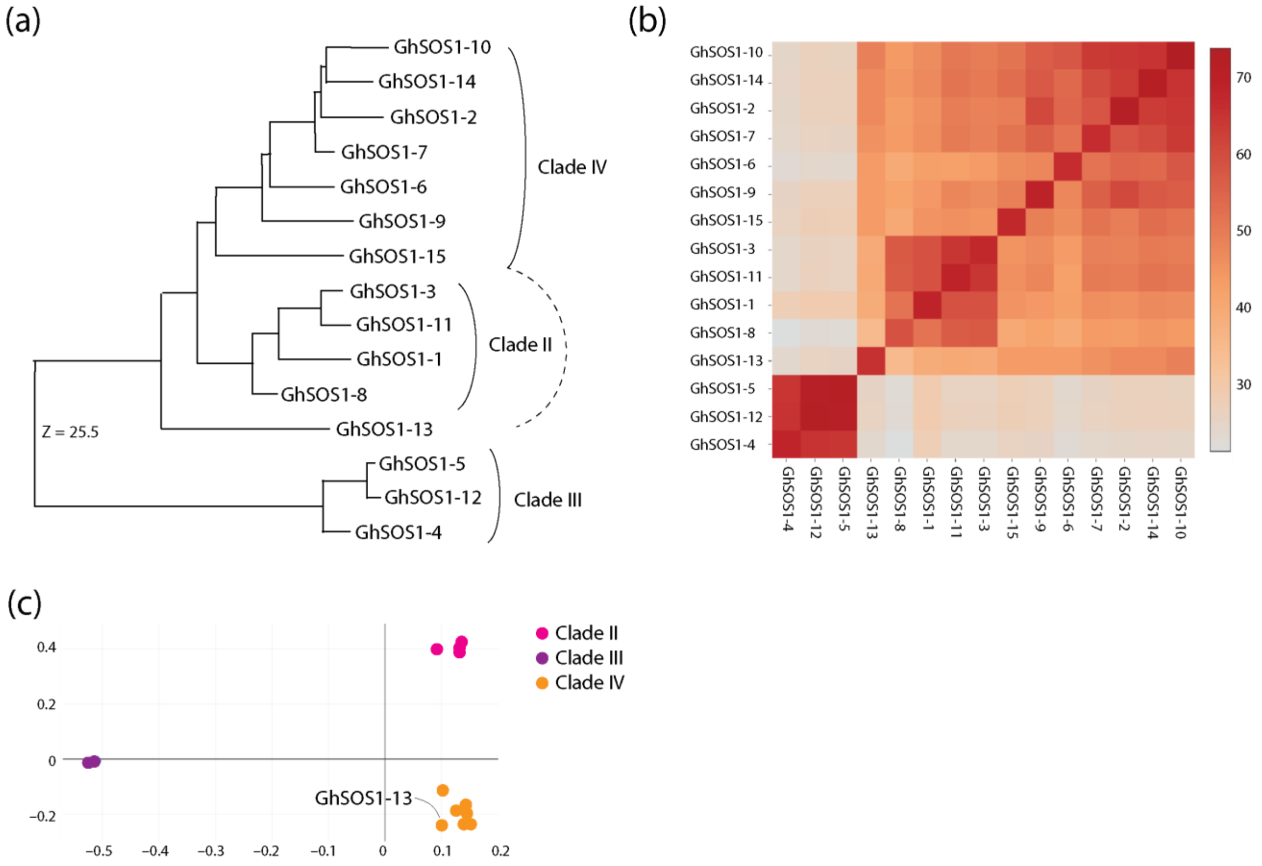

Figure S1. Structural similarity dendrogram (a), hierarchically clustered similarity matrix of Z-scores (b), and projections of the multidimensional scaling method of correspondence analysis (c) of all-against-all pairwise DALI structural alignments of AlphaFold2-predicted GhSOS1 proteins.
