# Supplementary material for: Genome-Wide Identification and Abiotic Stress-Responsive Expression Analysis of the SOS1 Gene Family in Gossypium hirsutum L
Source: Life (Basel). 2025 Nov 30;15(12):1843. doi: 10.3390/life15121843 (PMC12735070; doi:10.3390/life15121843)
Supplement: Supplementary file 1 [file life-15-01843-s001.zip › Figure S2.pdf]

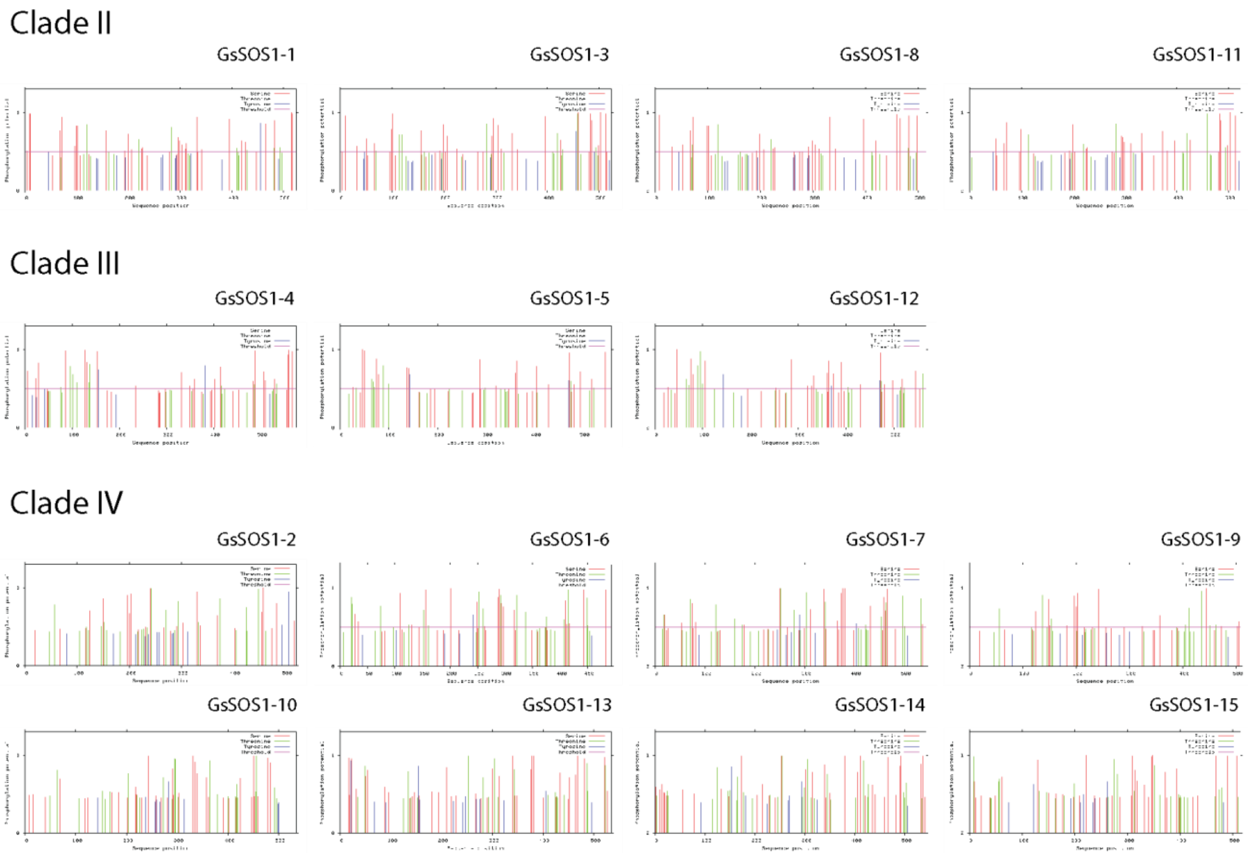

Figure S2. Phosphorylation sites in GhSOS1 proteins predicted using Net Phos 3.1. Most of the proteins have high levels of serine and threonine content indicating their potential role in phosphorylation.
