# Supplementary material for: Genome-Wide Identification and Abiotic Stress-Responsive Expression Analysis of the SOS1 Gene Family in Gossypium hirsutum L
Source: Life (Basel). 2025 Nov 30;15(12):1843. doi: 10.3390/life15121843 (PMC12735070; doi:10.3390/life15121843)
Supplement: Supplementary file 1 [file life-15-01843-s001.zip › Table S1.pdf]

life

MDPI

Table S1. Primer sequences

			Primers for expression study	
**S. No**	**Transcripts/Genes**	**Indexing****	**Forward primer (5’ to 3’)**	**Reverse primer (5’ to 3’)**
**1**	*GhUBQ14**	DQ116441	CAACGCTCCATCTTGTCCTT	TGATCGTCTTTCCCGTAAGC
**2**	*GhSOS1-4*	Gohir.A07G039800.1	GGTGGTGCTATCATACGCGA	GTCGCCGACGAATTCACAAG
**3**	*GhSOS1-5*	Gohir.A02G103100.1	AACAGCAGTTTTGGCAGCAG	CTAGGGCACCAGCAAATGGA
**4**	*GhSOS1-10*	Gohir.D01G209600.1	CCAACAACACGATACGGTGC	TTGATATCTCCCCCGTGCCT
**5**	*GhSOS1-11*	Gohir.D06G166000.1	CCAACAACACGATACGGTGC	TTGATATCTCCCCCGTGCCT
**6**	*GhSOS1-13*	Gohir.D11G077600.1	TGGATCGTGGACAAACTGGG	GCCGCCAAAGAAGAAGGTTG

* Housekeeping ** GeneBank accession no. / Phytozome Gene ID version 3.1
