# Supplementary material for: Genome-Wide Identification and Abiotic Stress-Responsive Expression Analysis of the SOS1 Gene Family in Gossypium hirsutum L
Source: Life (Basel). 2025 Nov 30;15(12):1843. doi: 10.3390/life15121843 (PMC12735070; doi:10.3390/life15121843)
Supplement: Supplementary file 1 [file life-15-01843-s001.zip › Table S2.pdf]

life

MDPI

Table S2. GhSOS1 protein sequences in FASTA format

>GhSOS1-1
MGISPAESGSPEKEQQAAGVGILLQIMMLVLSFVLGHVLRRHKFYYLPEASASLLIGLIVGGLANISNTETSIRAWFNFHE
EFFFLFLLPPIIFQSGFSLSAKPFFSNFGAIVTFAILGTFIASVVTGVLVYIGGRMYLMYGLPFVECLMFGALISATDPVTVLSI
FQELGTDTNLYALVFGESVLNDAMAISLYRTMSIVRKHASSAQNFFMVIFRFLETFVGSMSAGVGVGFVSALLFKYAGL
DVDNLQNLECCLFVLFPYFSYMLAEGLGLSGIVSILFTAIVMKHYSYSNLSENSQQFVSDFFHLISSLAETFTFIYMGFDIA
MEKHSWSHLGFIFFSIIFIVVARAANVFSCAYLVNLVRPVHRQIPLKHQKALWYSGLRGAMAFALALQSVHDLPEGRG
QIIFTATTAIVVLSVLLIGGSTGTMLEALHVVGDGHDSHLGESFDVNNGYVAPSFKEDGTSGNGIKMKLKEFHKRTSSFT
ALDRNYLTPFFTSQNEDDEGGEALLDERISSSKRGGFLGQ*

>GhSOS1-2
MFEFVRNLAHEHEQVVPISVFVAILCLCLVIGHLLEENRWVNESITAILIGGIAGTVILFLNKGKSSHILRFSEELFFIYLLPP
IIFNAGFQVKKKQFFQNFITIMLFGVIGVFISTSIITAGSWWLFPKLGFFGLTAREYLAVGTIFSSTDTVCTLQVLHQDENPL
LYSLVFGEGVVNDATSVVLFNAIQKIDVSRINSRTSFQLIGDFIYLFSTSTALGVTFGLVTAYSLKTLYFGRHSTVRELAIMV
LMAYLSYMLAELLDLSGILTVFFCGILMSHYAWYNVTESSRITTRHIFAMMSFVAETFIFLYVGMDALDMEKWKMTRLS
VGTLMASFGTLVFLILVGRAAFVFPLSAFSNCLNKHPDRSKPLTFRHQVVIWWAGLMRGAVSIALAFKQFTFSGVTWD
PVNAAMIANTIIVVLFTTLVFGFLTKPLILCLLPQHVTDTSDEAQGSKSPKEDITLPLLSFEGSASTNILRAKDSLSMLIERP
VYTVHFYWRKFDDRYMRPIFGGPISSPPEC*

>GhSOS1-3
MDNSTAEKGSPGQEQQAAGVGILLQIMMLVLSFVVGHVLRRHKFYYLPEASASLLIGLIVGGLANISDTERSIRAWFNF
HEEFFFLFLLPPIIFQSGFSLSPKPFFSNFGAIVTFAIFGTFIASVVTGVLVYLGGLMYLMYKLPFVECLMFGALISATDPVTV
LSIFQELGTDMNLYALVFGESVLNDAMAISLYRTMSVVRSNDPSGQNFFMVIVRFLETFVGSMSAGVGVGFTSALLFKY
AGLDIDNLQNLECCLFVLFPYFSYMLAEGLGLSGIVSILFTGIVMKHYTFSNLSENSQRFVSDFFHLISSLAETFIFIYMGFD
IAMEKHSWSHVGFIFFSILFIAIARAVNVFSCAYLINLVRPAHRQIPSKHQKALCYSGLRGAMAFALALQSVHDLEEGH
GQIIFTATTAIVVLTVLLIGGSTGTMLEALQVVGDGHDAHLGEGFEGNNGYVPTSREEDETTGNKLRMKLKEFHRSAAS
FSEIDRNYLTPFFTSQNGDSEDEEDDPMPSSRRGVYHGHS*

>GhSOS1-4
MAICSSRRGVLGSVYCLVVVLSYARICLSARSDNEIRERFYGNLVNSSATGTGEGSIAKMFDRVLEKEFSENDQTEGTDA
SNFNTSVADQQAVLETVAKITHEKVKRNDTQETNGTRAFQIQDVFSLENEDSDETTTLIDKKDNVFVMSNRKSKYPVL
QVDLRLISDLVVVIVSAAIGGIIFSCLGQPVIVGYLLAGSLIGPGGLKFISEMVQVETVAQFGVVFLLFALGLEFSLAKLKV
VGPVAVFGGLLQIVIFMCLCGIIAVLCGANLSEGVFVGSFLSMSSTAIVVKFLVERSSTNSLHGQVTIGTLIFQDCAVGLLF
ALLPVLGGSSGWLHGMVSMGKLILVLSIYLTIASLLSWSFVPRFLKLMMQISSQTNELYQLAAVAFCLLSAWCSDKMGL
SLELGSFVAGVMISTTDFAQHTLDQVEPIRNLFAALFLSGIGMLIHVHFLWSHVDILLASVILVIVVKTAVVCVVAKAFG
YSVRTSFHVGVLLAQIGEFAFVLLSRASNLHLVEGKMYLLLLGTTALSLVTTPLMFKLIPYVMNLGVLLQWFPSESSSTN
EEKVSIIEAHNRLH*

>GhSOS1-5
MREFSALFFICDLIVFITSVAVIDARSAVEINVTAVLANVSDPRSREDSFVGMIDRALEKEFNDTDQTEATDPDSFNNSVA
GKQAVLETVARVKTKKNETKEEKSFQLHDVFHLDDENRADDAPTLIDRNDNVFIISNPKSKYPVLQLDLRLILDLIIVIVS
ATCGGIAFACAGQPVITGYLLAGSIIGPGGFSFVGEMVQVETVAQFGVIFLLFALGLEFSATKLRVVRAVAVLGGLLQIFL
FMCLCGITVSLCGGKASEGVFVGAFLSMSSTAVVLKFLMERNSISALHGQVTIGTLILQDCAVGLLFALLPVLGGNSGVL
QGVLSMTKSLVVLITFLTILTIVSWTCVPWFLKLMISLSSQTNELYQLASVAFCLLVAWCSDKLGLSLELGSFAAGVMIST
TDLGQHTLEQVEPIRNFFAALFLASIGMLINVHFLWNHVDILLAAVILVIIIKTMVVAAVVKGFRYSNKTSILVGMSLAQI
GEFAFVLLSRASNLHLVEGKLYLLLLGTTALSLVTTPLLFKLIPAVVRLGVLLRWFPADSPEVHKRAKKQVD*

>GhSOS1-6
MNESITALAIGVCTGVIILLTTGGKSSRLLVFSEDLFFIYLLPPIIFNAGFQVKKKQFFRNFLTIMLFGAVGTLISFCVISIGAI
HFFKKMNIGDLTLGDYLAIGAIFSATDSVCTLQVLNQDETPLLYSLVFGEGVVNDATSVVLFNAIQTFDISHIDSTMALQ
YIGNFLYLFILSTLLGVLAGLLSAYIIRKLYFGRHSTDREVALMMLMAYLSYMLAELFSFSAILTVFFCGIVMSHYTWHNV
TESSRVTTKHAFATLSFVSEIFIFLFVGMDALDIEKWRVISDSPGTSVGVSGILLGLILVGRAAFVFPLSFISNLTRKAPHEK
VDFKQQVTVWWAGLMRGAVSMALAYNQFTSLGHTQLRGNAMMITSTISVVLFSTVVFGLMTKPLVRLLLPSPKHLMR
TLSSEPSTPKSFIVPLLGNEGEPEADQNNRNACRPTSLRMLLTTPSNTVHYYWRKFDDAFMRPVFGGRGFVPFVPGSPTE
QNGHQWQ*

>GhSOS1-7
MVAPQLAAVFTKLQTLSTSDHASVVSMNIFVALLCACIVIGHLLEENRWMNESITALIIGVFTGVIILLTSGGKSSHLLVF
SEDLFFIYLLPPIIFNAGFQVKKKQFFRNFITIMLFGAVGTLISCTIISLGVINFFKEMDIGSLDIGDFLAIGAIFAATDSVCTL
QVLNQDETPLLYSLVFGEGVVNDATSVVLFNAIQSFDLVNTSPRILLEFIGSFLYLFLASTMLGVIVGLVSAYIIKKLYFGR
HSTDREFALMMLMAYLSYIMAELFYLSGILTVFFCGIVMSHYTWHNVTESSRVTTKHAFATLSFVAETFLFLYVGMDAL
DMEKWRFVSDSPGTSVAVSAVLMGLVMVGRAAFVFPLSFLSNLAKKSTSEKISFREQIIIWWAGLMRGAVSMALAYNQ
FTRGGHTQLRGNAIMITSTITIVLFSTVVFGLMTKPLIRFLLPHPKPTASMLSDQSTPKSMEAPFLGSGQDSFDDSLIGVHR
PNSIRALLTTPAHTVHYYWRKFDNAFMRPMFGGRGFVPFVPGSPTERSEPNLPQWQ*

>GhSOS1-8
MGISPEESGSPEKEQQAAGVGILLQIMMLVLSFVLGHVLRRHKFYYLPEAGASLLIGLIVGGLANISNTETSIRAWFNFHE
EFFFLFLLPPIIFQSGFSLSAKPFFSNFGAIVTFAILGTFIASVVTGVLVYLGGRMYLMYGLPFVECLMFGALISATDPVTVLS
IFQELGTDTNLYALVFGESVLNDAMAISLYRTMSIVRKHASSAQNFFMVIFRFLETFVGSMSAGVGVGFVSALLFKYAGL
DVDNLQNLECCLFVLFPYFSYMLAEGLGLSGIVSILFTAIVMKHYSYSNLSENSQQFVSDFFHLISSLAETFTFIYMGFDIA
MEKHSWSHLGFIFFSILFIVVARAANVFSCAYLVNLVRPVHRQIPLKHQKALWYSGLRGAMAFALALQSVHDLPEGRG
QIIFTATTAIVVLSVLLIGGSTGTMLEALHVVGDSHDGHLGESFDVNNGYVAPSFKKDGTSGNGIKMKLKEFHKRTSSFT
ALDRNYLTPFFTSQNEDDEEEEEALLDERI*

>GhSOS1-9
MFEFVRNLAHEHEQVVPISVFVAILCLCLVIGHLLEENRWVNESITAILIGGVAGTVILFLNKGKSSHILRFSEELFFIYLLP
PIIFNAGFQVKKKQFFQNFITIMLFGVIGVFISTSIITAGSWWLFPKLGFFGLTAREYLAVGTIFSSTDTVCTLQVLHQDENP
LLYSLVFGEGVVNDATSVVLFNAIQKIDVSRINSRTSLQLIGDFIYLFSTSTALGVTFGLVTAYSLKTLYFGRHSTVRELAIM
VLMAYLSYMLAELLDLSGILTVFFCGILMSHYAWHIFAMMSFVAETFIFLYVGMDALDMEKWKMTRLSVGTLMASFGT
LVFLILVGRAAFVFPLSAFSNYLNKHPDRSKPLTFRHQVVIWWAGLMRGAVSIALAFKQFTFSGVTWDPVNAAMITNTI
IVVLFTTLVFGFLTKPLILCLLPQHVTDTSDEGQGSKSPKEDMTLPLLSFEASASTNILRAKDSLSMLIERPVYTVHFYWRK
FDDRYMRPIFGGPISSPPEC*

>GhSOS1-10
MAIGILNSLLASDHSSIVSMNLFVALLCGCIVIGHLLEESRWMNESITALVIGVCTGVVILLTTGGKSSHLLVFSEDLFFIYL
LPPIIFNAGFQVKKKQFFRNFMTIMLFGAVGTLISFGIISAGAIQFFKELHIGDLQIGDYLAIGAIFSATDSVCTLQVLNQD
ETPLLYSLVFGEGVVNDATSVVLFNAIQSFDLNHINSTIALKFVGNFFYLFISSTLLGVVTGLLSAFIIKKLYFGRHSTDREV
ALMILMAYLSYMLAELFYLSGILTVFFCGIVMSHYTWHNVTESSRVTTKHAFATLSFVAEIFIFLYVGMDALDIEKWRVIS
DSPGKSVGVSSILLGLILVGRAAFVFPLSFISNLTKKAPHEKIEFKQQVTIWWAGLMRGAVSMALAYNQFTSLGHTQVR
GNAMMITSTITVVLFSTVVFGLMTKPLVRILLPSPKHLSRMLSSEPTTPKSFFLPLLNNGQESEAEQGNRSVIRPSSLRMLL
TTPSHTVHYYWRKFDDAFMRPVFGGRGFVPFVPGSPTEQNGPQWQ*

>GhSOS1-11
MDNSTAEKGSPGQEQQAAGVGILLQIMMLVLSFVVGHVLRRHKFYYLPEASASLLIGLIVGGLANISDTERSIRAWFNF
HEEFFFLFLLPPIIFQSGFSLSPKPFFSNFGAIVTFAIFGTFIASVVTGVLVYLGGLMYLMYKLPFVECLMFGALISATDPVTV
LSIFQELGTDMNLYALVFGESVLNDAMAISLYRTMSVVRSNDPSGQNFFMVIVRFLETFVGSMSAGVGVGFTSALLFKY
AGLDIDNLQNLECCLFVLFPYFSYMLAEGLGLSGIVSILFTGIVMKHYTFSNLSENSQHFVSDFFHLISSLAETFIFIYMGFD
IAMEKHSWSHVGFIFFSILFIAIARAVNVFSCAYLINLVRPAHRQIPSKHQKALCYSGLRGAMAFALALQSVHDLQEGH
GQIIFTATTAIVVLTVLLIGGSTGTMLEALQVVGDGHDAHLGEGFEGNNGYVPTSREEDETTGNKLRMKLKEFHRSAAS
FSEIDRNYLTPFFTSQNGDSEDEEDDPMPSSRRGIYHGHS*

>GhSOS1-12
MRGICALFFICDLIVFITSVAVIDARSAVEINVTAVLANISDPRSREDSFVGMIDRALEKEFNDTDQNEATDPDSFNNSVA
GKQAVLETVARVKTKKNETKEEKSFQLHDVFHLDDENRADDAPTLIDRNDNVFIISNPKSKYPVLQLDLRLILDLIIVIVS
ATCGGIAFACAGQPVITGYLLAGSIIGPGGFSFVGEMVQVETVAQFGVIFLLFALGLEFSATKLRVVRAVAVLGGLLQIFL
FMCLCGITVSLCGGKASEGVFVGAFLSMSSTAVVLKFLMERNSISALHGQVTIGTLILQDCAVGLLFALLPVLGGNSGVL
QGVLSMTKSLVVLITFLTILTIVSWTCVPWFLKLMISLSSQTNELYQLASVAFCLLVAWCSDKLGLSLELGSFAAGVMIST
TDLGQHTLEQVEPIRNFFAALFLASIGMLINVHFLWNHVDILLAAVILVIIIKTMVVAAVVKGFRYSNKTSILVGMSLAQI
GEFAFVLLSRASNLHLVEGKLYLLLLGTTALSLVTTPLLFKLIPAVVRLGVLLRWFPADSPEIGLKGDSLRVFGRTARSVL
L*

>GhSOS1-13
MVAPHLAAVFTKLQTLSISDYASVENRWMNESITALIIGVFTGVIILLTSGGKSSHLLVFSEDLFFIYLLPPIIFNAGFVLFKY
PFLSLYSGFNLSALRIPQCFHCCKQRNIDVKKKQFFRNFITIMLFGAVGTLISCTIISLGISNDLMETIYSIYQLAKMELLLM
MQVLNQDEPPLLYSLVFGEGVVNDATSVVLFNAIQSFDLVNTSPRILLEFIGSFLYLFLASTMLGVIVGLVSAYIIKKLYFG
RHSTDREFALMMLMAYLSYIMAELFYLSGILTVFFCGIVMSHYTWHNVTESSRVTTKHAFATLSFVAETFLFLYVGMDA
LDTEKWRFVSDSPGTSVAVSAVLMGLVMVGRAAFVFPLSFLSNLAKKSASEKISFREQIIIWWAGLMRGAVSMALAYN
QFTRGGHTQLRGNAIMITSTITIVLFSTVVFGLMTKPLISMLSDQSTPKSMEAPFLGSGQDSFDDSLIGVHRPNSIRVLLTT
PAHTVHYYWRKFDNAFMRPMFGGRGFVPFVPGSPTERSEPNLPQWQ*

>GhSOS1-14
MSTIEIETMSHLSTLQSSSDFLSTTTIIALTIFFSLLCACIIIGHLLEENRWANESITALLLGLCAGAVVLLASKGNSSKILVFS
EDLFFLYLLPPIIFNAGFQVKKKQFFKNFTIILMFGIFGTVISFCLISVGAFLLFNRIGVTSLNTQDYLAVGAILSATDTVCTL
QVLSQDETPFLYSVIFGEGVVNDATSIVLFNAVQSLDFNNIDAMISLKLLGTFLYLFFTSTILGVVAGLLSAFIIKTLYFGRH
STDREVALMMIMAYLSYMLAELLNLSGILTVFFCGIVMSHYTWHNVTQSSRITTKHAFATMSFIAETFIFLYVGMDALDI
DKWKASSASAGTFIAVSSTLFALVLVGRAAFVYPLANFINCIRKRDGSNIAFRKQFIMWWAGLMRGAVTIALSYNQFSN
SDDEDTQDSALMITSTIIVVLFSTVVFGSITKPLIEAVLLRHAKPNVSDATDIPSLDDLRLMFIENGEPSDMGAGPRRSSLR
LLLTHPTWTVHYLWRKFDDRFMRPVFGGRGFVPFVPGSPTGASDEASRT*

>GhSOS1-15
MALDPDQTSVDSITLFVALLFSCIVIGHLLEKNRWFNESTTALAIGLCSGIIILLTTEGKRSHILVFNEELFFIYLLPPIIFNAG
FQVKKKQFFRNFAMIILFGTMQLFKKLDIGFLDIGDYLAIGAIFSATDSVCTLQVLNQDETPLLYSIVFGEGVVNDATSIV
LFNAIQKFDLSHITLRIFIEFIGNFLYFFITSTLLGVGVGLISAYIIKKLYIGRHSTDREVALMILMAYLSYMMAELFNLSSILT
VFFCGIVMSHYTWHNVTESSRITTKHAFATLSFISEIFIFLYVGMDALDIEQWKVVSKSPGTSAGVSSILLGLVLVGRAASV
FPLSFISNLFKRSESDKFTLKQQFVKQVTIWWAGLMRGSVSVALAYNQFTRSGHTQLRGNSIMITSTITVVLFSTVVFGLM
TKPLIRLLLPSKHLCGGVSSGSFDSFPSKLMTDLPLIANGDAEVGGNNIPRPTSLRMLLATPTRTVHYYWRKFDDSVMRP
MFGGRGFMPHVPGSPTEPLLH*
