# Supplementary material for: Genome-Wide Identification and Abiotic Stress-Responsive Expression Analysis of the SOS1 Gene Family in Gossypium hirsutum L
Source: Life (Basel). 2025 Nov 30;15(12):1843. doi: 10.3390/life15121843 (PMC12735070; doi:10.3390/life15121843)
Supplement: Supplementary file 1 [file life-15-01843-s001.zip › Table S3.pdf]

life

MDPI

Table S3. Selection pressure analysis. SD: Segmental duplication, TD: Tandem duplication.

Paralog 1	Paralog 2	Ks	Ka	Ka/Ks	Duplication	Evolution
GhSOS1-1	GhSOS1-2	2.127	0.939	0.44	TD	Negative
GhSOS1-1	GhSOS1-3	0.34	0.078	0.23	SD	Negative
GhSOS1-1	GhSOS1-4	1.754	1.595	0.91	SD	Negative
GhSOS1-1	GhSOS1-5	2.028	1.371	0.68	SD	Negative
GhSOS1-1	GhSOS1-6	1.974	1.361	0.69	SD	Negative
GhSOS1-1	GhSOS1-8	2.287	1.545	0.68	SD	Negative
GhSOS1-1	GhSOS1-9	0.036	0.016	0.44	SD	Negative
GhSOS1-1	GhSOS1-12	3.171	0.955	0.30	SD	Negative
GhSOS1-1	GhSOS1-15	2.277	0.976	0.43	SD	Negative
GhSOS1-1	GhSOS1-14	2.147	0.987	0.46	SD	Negative
GhSOS1-2	GhSOS1-3	2.844	0.905	0.32	SD	Negative
GhSOS1-2	GhSOS1-4	3.16	1.615	0.51	SD	Negative
GhSOS1-2	GhSOS1-5	2.388	1.35	0.57	SD	Negative
GhSOS1-2	GhSOS1-6	2.657	1.357	0.51	SD	Negative
GhSOS1-2	GhSOS1-8	2.598	1.601	0.62	SD	Negative
GhSOS1-2	GhSOS1-9	2.141	0.938	0.44	SD	Negative
GhSOS1-2	GhSOS1-12	2.307	0.33	0.14	SD	Negative
GhSOS1-2	GhSOS1-13	1.868	1.536	0.82	SD	Negative
GhSOS1-2	GhSOS1-15	2.536	0.351	0.14	SD	Negative
GhSOS1-3	GhSOS1-4	1.904	1.653	0.87	SD	Negative
GhSOS1-3	GhSOS1-5	2.016	1.389	0.69	SD	Negative
GhSOS1-3	GhSOS1-6	2.364	1.328	0.56	SD	Negative
GhSOS1-3	GhSOS1-8	2.63	1.576	0.60	SD	Negative
GhSOS1-3	GhSOS1-9	0.347	0.069	0.20	SD	Negative
GhSOS1-3	GhSOS1-13	2.682	1.629	0.61	SD	Negative
GhSOS1-3	GhSOS1-15	2.415	0.913	0.38	SD	Negative
GhSOS1-3	GhSOS1-14	2.322	0.925	0.40	SD	Negative
GhSOS1-3	GhSOS1-4	1.904	1.653	0.87	SD	Negative
GhSOS1-4	GhSOS1-8	2.491	0.182	0.07	SD	Negative
GhSOS1-4	GhSOS1-9	1.67	1.618	0.97	SD	Negative
GhSOS1-4	GhSOS1-12	1.739	1.708	0.98	SD	Negative
GhSOS1-4	GhSOS1-15	1.808	1.64	0.91	SD	Negative
GhSOS1-4	GhSOS1-14	1.609	1.636	1.02	SD	Positive
GhSOS1-5	GhSOS1-6	0.207	0.046	0.22	TD	Negative
GhSOS1-5	GhSOS1-9	1.998	1.334	0.67	SD	Negative
GhSOS1-5	GhSOS1-10	3.178	0.374	0.12	SD	Negative
GhSOS1-5	GhSOS1-12	1.616	1.351	0.84	SD	Negative
GhSOS1-5	GhSOS1-13	2.684	0.879	0.33	SD	Negative

GhSOS1-5	GhSOS1-15	1.95	1.375	0.70	SD	Negative
GhSOS1-5	GhSOS1-14	3.064	1.333	0.44	SD	Negative
GhSOS1-6	GhSOS1-8	2.396	1.732	0.72	SD	Negative
GhSOS1-6	GhSOS1-9	1.918	1.329	0.69	SD	Negative
GhSOS1-6	GhSOS1-12	1.796	1.341	0.75	SD	Negative
GhSOS1-6	GhSOS1-15	1.997	1.385	0.69	SD	Negative
GhSOS1-7	GhSOS1-11	3.138	0.726	0.23	SD	Negative
GhSOS1-7	GhSOS1-13	2.833	0.949	0.34	SD	Negative
GhSOS1-7	GhSOS1-15	2.362	1.485	0.63	SD	Negative
GhSOS1-8	GhSOS1-9	2.127	1.555	0.73	SD	Negative
GhSOS1-8	GhSOS1-12	1.842	1.823	0.99	SD	Negative
GhSOS1-8	GhSOS1-13	2.316	1.878	0.81	SD	Negative
GhSOS1-8	GhSOS1-15	2.022	1.785	0.88	SD	Negative
GhSOS1-9	GhSOS1-15	2.519	0.965	0.38	SD	Negative
GhSOS1-9	GhSOS1-14	2.258	0.967	0.43	SD	Negative
GhSOS1-10	GhSOS1-11	2.662	0.816	0.31	SD	Negative
GhSOS1-10	GhSOS1-14	2.244	1.445	0.64	SD	Negative
GhSOS1-11	GhSOS1-12	2.751	1.808	0.66	SD	Negative
GhSOS1-11	GhSOS1-13	2.27	1.021	0.45	SD	Negative
GhSOS1-11	GhSOS1-15	2.582	1.714	0.66	SD	Negative
GhSOS1-12	GhSOS1-13	2.276	1.466	0.64	SD	Negative
GhSOS1-12	GhSOS1-15	0.491	0.16	0.33	SD	Negative
GhSOS1-13	GhSOS1-15	2.599	1.518	0.58	TD	Negative
GhSOS1-13	GhSOS1-14	1.464	1.551	1.06	TD	Positive
