# Supplementary material for: Genome-Wide Identification and Abiotic Stress-Responsive Expression Analysis of the SOS1 Gene Family in Gossypium hirsutum L
Source: Life (Basel). 2025 Nov 30;15(12):1843. doi: 10.3390/life15121843 (PMC12735070; doi:10.3390/life15121843)
Supplement: Supplementary file 1 [file life-15-01843-s001.zip › Table S4.pdf]

life

MDPI

Table S4. Confidence of Phyre 2.2 and AlphaFold2 predictions and TM-scores of structural alignments

GhSOS1 protein	Phyre 2.2 Confidence (%)	Phyre 2.2 Coverage (%)	Phyre 2.2 Top template (PDB entry)	AlphaFold2 Confidence*	Alignment TM-score
**GhSOS1-1**	100	73	Na^+/H^+ exchanger (6z3z_A)	79.4 (0.763)	0.647
**GhSOS1-2**	100	86	Na^+/H^+ exchanger (8pvr_A)	78.9 (0.798)	0.802
**GhSOS1-3**	100	86	Na^+/H^+ exchanger (8pvr_A)	77.8 (0.773)	0.799
**GhSOS1-4**	100	66	K^+ efflux system (8by2_A)	78.5 (0.710)	0.545
**GhSOS1-5**	100	68	K^+ efflux system (8by2_A)	77.4 (0.708)	0.563
**GhSOS1-6**	100	84	Na^+/H^+ exchanger (8pvr_A)	77.7 (0.787)	0.768
**GhSOS1-7**	100	82	Na^+/H^+ exchanger (8pvr_A)	77.2 (0.786)	0.777
**GhSOS1-8**	100	88	Na^+/H^+ exchanger (8pvr_A)	79.1 (0.786)	0.806
**GhSOS1-9**	100	87	Na^+/H^+ exchanger (8pvr_A)	77.8 (0.766)	0.767
**GhSOS1-10**	100	83	Na^+/H^+ exchanger (8pvr_A)	77.7 (0.796)	0.784
**GhSOS1-11**	100	86	Na^+/H^+ exchanger (8pvr_A)	77.8 (0.777)	0.798
**GhSOS1-12**	100	67	K^+ efflux system (8by2_A)	76.6 (0.710)	0.556
**GhSOS1-13**	100	81	Na^+/H^+ exchanger (8pvr_A)	66.5 (0.723)	0.673
**GhSOS1-14**	100	82	Na^+/H^+ exchanger (8pvr_A)	78.6 (0.801)	0.780
**GhSOS1-15**	100	84	Na^+/H^+ exchanger (8pvr_A)	75.4 (0.709)	0.771

*Average pLDDT score (predicted TM-core)
