# Supplementary material for: Genome-Wide Identification and Abiotic Stress-Responsive Expression Analysis of the SOS1 Gene Family in Gossypium hirsutum L
Source: Life (Basel). 2025 Nov 30;15(12):1843. doi: 10.3390/life15121843 (PMC12735070; doi:10.3390/life15121843)
Supplement: Supplementary file 1 [file life-15-01843-s001.zip › Table S5.pdf]

life

MDPI

Table S5. Structural alignment of the SOS1 Na^+/H^+ antiporter of *A. thaliana* (PDB entry 8JD9) and the GhSOS1-1 protein

```
US-align v. 20241108
Name of Structure_1: 8JD9.pdb:A
Name of Structure_2: GhSOS1-1.pdb:A

Aligned length= 389
RMSD =3.24
Sequence identity = 0.195
TM-score = 0.38412
(normalized by length of Structure_1: L=935, d0=10.26)
TM-score = 0.82488
(normalized by length of Structure_2: L=411, d0=7.31)

":" denotes residue pairs of d < 5.0 Å
"." denotes other aligned residues

         10        20        30        40        50        60
         |         |         |         |         |         |
KLESSPVDAVLFVGMSLVLGIASRHLLRGTRV------PYTVALLVIGIALGSLEYGAKH
         ::::::.:::::::::....         .::::.::..::..:::::::.
---------QIMMLVLSFVLGHVLRRHKF---YYLPEASASLLIGLIVGGLANISNTETS

         70        80        90       100       110       120
         |         |         |         |         |         |
NLGKIGHGIRIWNEIDPELLLAVFLPALLFESSFSMEVHQIKRCLGQMVLLAVPGVLIST
.    ..:::::  : ::::: :::::::::::::::::::::::::::::: :::::::
I----RAWFNFH--E-EFFFL-FLLPPIIFQSGFSLSAKPFFSNFGAIVTFA-ILGTFIA

        130       140       150       160       170       180
         |         |         |         |         |         |
ACLGSLVKVTF-PY----EWDWKTSLLLGGLLSATDPVAVVALLKELGASKKLSTIIEGE
::::::::::: ..    ::::::::::::::::::.::::::::::.::::::::::::
SVVTGVLVYIGGRMYLMYGLPFVECLMFGALISATDPVTVLSIFQELGTDTNLYALVFGE

        190       200       210       220       230       240
         |         |         |         |         |         |
SLMNDGTAIVVFQLFLKMAMGQNS--DWSSIIKFLLKVALGAVGIGLAFGIASVIWLK-F
:::::::::::::::::.......  ...::::::::::::::::::::::::::::: :
SVLNDAMAISLYRTMSIVRKHASSAQNFFMVIFRFLETFVGSMSAGVGVFVSALLFKYA

        250       260       270       280       290       300
         |         |         |         |         |         |
---IFNDTVIEITLTIAVSYFAYYTAQEWAGASGVLTVMTLGMFYAAFARTAFKGDSQKS
   :::::::::::::::::::::::::::::::::::::: ::::::::::.:::::::
GLDVDNLQNLECCLFVLFPYFSYMLAEGLGLSGIVSILFTA-IVMKHYSYSNLSENSQQF

        310       320       330       340       350       360
         |         |         |         |         |         |
LHHFWEMVAYIANTLIFILSGVVIAEGILDSDKIAYQGNSWRFLFLLYVYIQLSRVVVVG
::::::::::: ::::::::::::::: ::..::: : :   ::::::::::::::::::
VSDFFHLISSL-AETFTFIYMGFDIAM-EKHSWSH-L-G---FIFFSIIFIVVARAANVF

        370       380       390       400       410       420
         |         |         |         |         |         |
VLYPLLCRFG-----YGLDWKESIILVWSGLRGAVALALSLSVKQSSGNSHISKETGTLF
::::::::..     .:::::::::: :: ::::::::::: : ::.  ..::.::::::
SCAYLVNLVRPVHRQIPLKHQKALWY-SG-LRGAMAFALAL-Q-SVH--DLPEGRGQIIF

        430       440       450       460       470       480
         |         |         |         |         |         |
LFFTGGIVFLTLIVNGSTTQFVLRLLRMDILPAPKKRILEYTKYEMLNKALRAFQDLGDD
::::::::::::: :::::::::::: :
TATTAIVVLSVLL-IGGSTGTMLEAL-H--------------------------------

        490       500       510       520       530       540
         |         |         |         |         |         |
EELGPADWPTVESYISSLKGSLDPKSLKDIRMRFLNGVQATYWEMLDEGRISEVTANILM

------------------------------------------------------------

        550       560       570       580       590       600
         |         |         |         |         |         |
QSVDEALDQVSTTLCDWRGLKPHVNFPNYYNFLHSKVVPRKLVTYFAVERLESACYISAA

------------------------------------------------------------

        610       620       630       640       650       660
         |         |         |         |         |         |
FLRAHTIARQQLYDFLGESNIGSIVINESEKEGEEAKKFLEKVRSSFPQVLRVVKTKQVT

------------------------------------------------------------

        670       680       690       700       710       720
         |         |         |         |         |         |
YSVLNHLLGYIENLEKVGLLEEKEIAHLHDAVQTGLKKLLRNPPIVKLPKLSDMITSHPL

------------------------------------------------------------

        730       740       750       760       770       780
         |         |         |         |         |         |
SVALPPAFCEPLKHSKKEPMKLRGVTLYKEGSKPTGVWLIFDGIVKWKSKILSNNHSLHP

------------------------------------------------------------

        790       800       810       820       830       840
         |         |         |         |         |         |
TFSHGSTLGLYEVLTGKPYLCDLITDSMVLCFFIDSEKILSLQSDSTIDDFLWQESALVL

------------------------------------------------------------

        850       860       870       880       890       900
         |         |         |         |         |         |
LKLLRPQIFESVAMQELRALVSTESSKLTTYVTGESIEIDCNSIGLLLEGFVKPVGIKEE

------------------------------------------------------------

        910       920       930       940       950
         |         |         |         |         |
LISSPAALSPSNATQYIVETRARAIIFNIHRGLMSWPENISLSERAMQLSIFGSMVN

---------------------------------------------------------
```
